# Hot exciton transition for organic light-emitting diodes: tailoring excited-state properties and electroluminescence performances of donor–spacer–acceptor molecules†

**DOI:** 10.1039/c8ra07891b

**Published:** 2018-11-06

**Authors:** Jayaraman Jayabharathi, Sekar Panimozhi, Venugopal Thanikachalam

**Affiliations:** Department of Chemistry, Annamalai University Annamalainagar 608 002 Tamilnadu India jtchalam2005@yahoo.co.in +91 9443940735

## Abstract

The photophysical, electrochemical and electroluminescent properties of newly synthesized blue emitters with donor–π–acceptor geometry, namely, 4′-(1-(naphthalen-1-yl)-1*H*-phenanthro[9,10-*d*]imidazol-2-yl)-*N*,*N*-diphenyl-(2-[1,1′-biphenyl]vinyl)-4-amine (NSPI-TPA), 4′-(1-(2-methylnaphthalen-1-yl)-1*H*-phenanthro[9,10-*d*]imidazol-2-yl)-*N*,*N*-diphenyl-(2-[1,1′-biphenyl]vinyl)-4-amine (MNSPI-TPA), 4-(2-(4′-(diphenylamino)-(2-[1,1′-biphenyl]vinyl)-4-yl)-1*H*-phenanthro[9,10-*d*]imidazol-1-yl)-1-naphthalene-1-carbonitrile (SPNCN-TPA) and 4-(2-(4-(9*H*-carbazol-9-yl)styryl)-1*H*-phenanthro[9,10-*d*]imidazol-1-yl)naphthalene-1-carbonitrile (SPNCN-Cz) were analyzed. The conjugation length in the emitters is not conducive to pure emission and hence, a molecular twisting strategy was adopted in NSPI-TPA, MNSPI-TPA, SPNCN-TPA and SPNCN-Cz to enhance pure emission. The emissive state (HLCT) of twisted D–π–A molecules containing both LE and CT (HLCT) states was tuned for high PL (*η*_PL_) (LE) and high exciton utilization (*η*_s_) (CT) efficiencies by replacing triphenylamine (strong donor) with carbazole (weak donor). Among strong donor compounds, namely, NSPI-TPA, MNSPI-TPA and SPNCN-TPA, the SPNCN-TPA-based device exhibited blue emission (451 nm) with CIE coordinates (0.15, 0.08), maximum current efficiency (*η*_c_) of 2.32 cd A^−1^, power efficiency (*η*_p_) of 2.01 lm W^−1^ and external quantum efficiency (*η*_ex_) of 3.02%. The device with SPNCN-Cz emitter exhibited higher electroluminescence efficiencies than the SPNCN-TPA-based device, with pure blue emission (443 nm, CIE: 0.15,0.07), *η*_ex_ of 3.15%, *η*_c_ of 2.56 cd A^−1^ and *η*_p_ of 2.45 lm W^−1^.

## Abstract 2

SPNCN-Cz device exhibits *η*_ex_ (3.15%), *η*_c_ (2.56 cd A^−1^), *η*_p_ (2.45 lm W^−1^) with CIE (0.15, 0.07).

## Graphical abstract

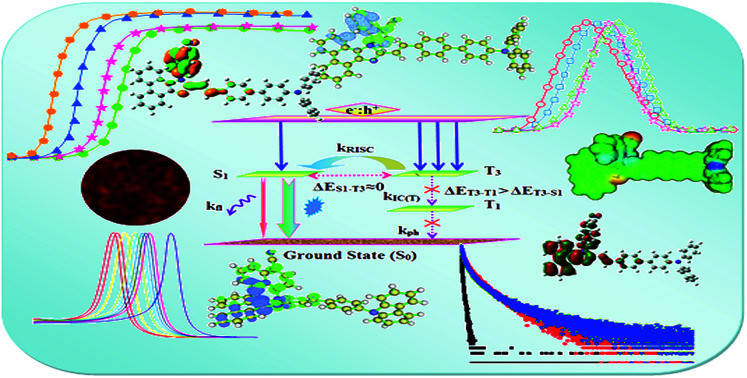

## 1 Introduction

**Scheme 1:**
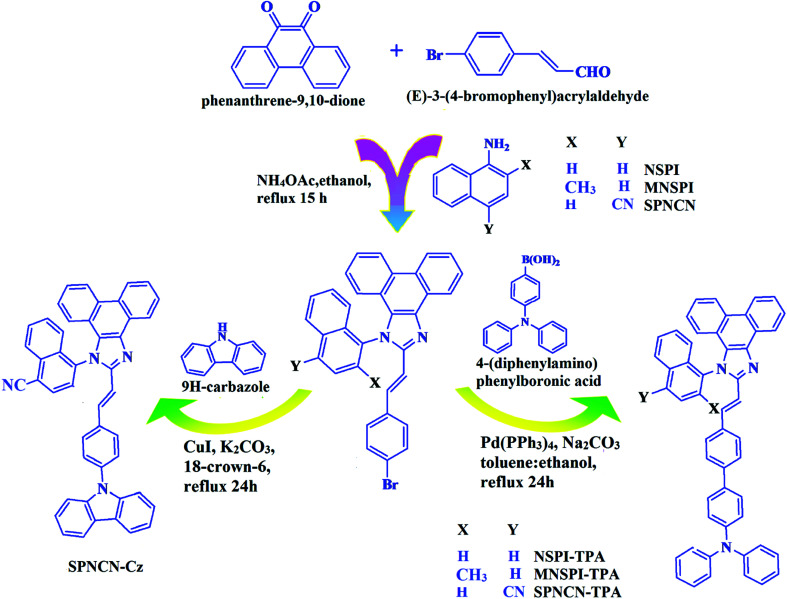
Synthesis routes of NSPI-TPA, MNSPI-TPA, SPNCN-TPA and SPNCN-Cz.

Organic light-emitting diodes (OLEDs) have been widely investigated and tested commercially in recent decades and utilized in flat-panel displays.^1^ However, their commercialization is still restricted because of the scarcity of blue emitters.^2–4^ Efficient green and red emissive materials are highly exploited. However, fabrication of blue OLEDs is a major problem due to wide band gap and unbalanced carrier injection.^5–10^ Therefore, the design of efficient pure blue emitters with narrow full width at half maximum (FWHM) is still an important task.^11,12^ The aggregated arrangement of the emitter-induced molecular interaction results in bathochromic shift with low quantum efficiency.^13^ However, non-doped blue emitters with restricted intermolecular interaction exhibit expected quantum yields.^14^ Their potential carrier injection and transporting abilities provide balanced charge recombination, which results in enhanced efficiency.^15–17^ For TV displays, blue OLEDs with CIE (0.14, 0.08) and (0.15, 0.06) are required by the National Television System Committee (NTSC) and high-definition television (HDTV), respectively. Pyrene^18^ and anthracene^19^ derivative-based blue OLEDs exhibit high efficiency with poor color purity. Therefore, to achieve pure blue emission, emitters having D–π–A geometry with twisted configuration are employed as they reduce the π-conjugation and consequently exhibit blue emission.^20–26^ Furthermore, the emissive state of twisted D–π–A molecules^27–30^ possesses both LE and CT states (hybridized local and charge transfer state/HLCT) and shows high PL efficiency (LE) and high exciton utilization (*η*_s_) (CT), which are attributed to hot exciton mechanism.^31–33^ The LE-dominated (low lying) HLCT state provided high radiative rate (*k*_r_), resulting in high photoluminescence efficiency (*η*_PL_) of the film, whereas CT-dominated HLCT state is responsible for high *η*_S_ through RISC *via* the hot exciton principle.^34^ The larger energy gap between T_2_ and T_1_ states greatly reduces the internal conversion (IC) 
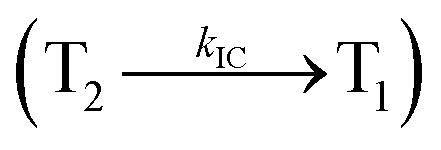
, resulting in hot RISC 
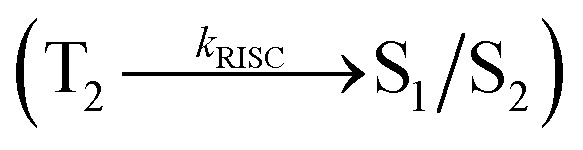
 rather than cold RISC (T_1_ → S_1_) TADF mechanism.^35,36^ Hot exciton process with HLCT increases external quantum efficiency (*η*_ex_) because of high *η*_PL_ and high *η*_S_. *η*_ex_ can be calculated as follows: *η*_ex_ = *η*_IQE_ × *η*_out_ = *η*_rec_ × *η*_PL_ × *η*_S_ × *η*_out_, where *η*_IQE_ is the internal quantum efficiency, *η*_out_ is the light out coupling efficiency (20%), *η*_rec_ is the efficiency for electron–hole recombination (100%), *η*_PL_ is the photoluminescence efficiency of the film and *η*_S_ is the exciton utilization efficiency [*η*_S_ = *η*_rec_ × *η*_PL_ × *η*_out_ ÷ *η*_EL_].^37^ The ambipolar phenanthrimidazole derivatives have been shown as potential blue emitters. The increase in conjugation in phenanthrimidazoles influenced the blue emission,^38–42^ and conjugation length was restricted by incorporating a bulky fragment in the core molecule, which resulted in twisted conformation.^43–46^ In line with this discussion and our own research interest, we report donor–spacer–acceptor derivatives, namely, 4′-(1-(naphthalen-1-yl)-1*H*-phenanthro[9,10-*d*]imidazol-2-yl)-*N*,*N*-diphenyl-(2-[1,1′-biphenyl]vinyl)-4-amine (NSPI-TPA), 4′-(1-(2-methylnaphthalen-1-yl)-1*H*-phenanthro[9,10-*d*]imidazol-2-yl)-*N*,*N*-diphenyl-(2-[1,1′-biphenyl]vinyl)-4-amine (MNSPI-TPA), 4-(2-(4′-(diphenylamino)-(2-[1,1′-biphenyl]vinyl)-4-yl)-1*H*-phenanthro[9,10-*d*]imidazol-1-yl)-1-naphthalene-1-carbonitrile (SPNCN-TPA) and 4-(2-(4-(9*H*-carbazol-9-yl)styryl)-1*H*-phenanthro[9,10-*d*]imidazol-1-yl)naphthalene-1-carbonitrile (SPNCN-Cz) using triphenylamine as a strong donor and carbazole as weak donor (Scheme 1). The H–H repulsion of bulky styryl fragment with the phenyl moiety of TPA and the carbazole moieties leads to twisted configuration, which enhances the twist angle, thus shortening the conjugation length. The solvatochromic effect of NPSI-TPA, MNSPI-TPA, SPNCN-TPA and SPNCN-Cz was examined to understand the excited state characteristics and interstate coupling strength of LE and CT components. Combining theoretical (TD-DFT) and experimental data, the LE and CT compositions were discussed using natural transition orbital (NTO), centroids of charges and transition density matrix (TDM) analysis. Hybridization of LE and CT energy states was used for molecular design, and their composition in HLCT was tuned, resulting in high EL efficiency. Triphenylamine (TPA) of SPNCN-TPA was replaced by carbazole (Cz) in SPNCN-Cz, both of which differ in electron donating ability. As a result, the CT composition decreases with an increase in LE composition in the S_1_ HLCT state. Thus, the SPNCN-Cz-based device exhibited maximum electroluminescent efficiency with *η*_ex_ of 3.15%, *η*_c_ of 2.56 cd A^−1^ and *η*_p_ of 2.45 lm W^−1^ and CIE of (0.15, 0.07), which were higher than those of the SPNCN-TPA-based device. Higher *η*_PL_ of SPNCN-Cz film was observed compared with that of SPNCN-TPA film; thus, high *η*_PL_ and high *η*_S_ enhanced the *η*_ex_ of the SPNCN-Cz-based device. These results can be used to design low cost fluorescent materials *via* subtle molecular modifications using the HLCT emissive state principle.

## 2 Experimental

### 2.1 Synthesis of *p*-bromostyrylphenanthroimidazoles (NSPI, MNSPI & SPNCN)

The synthesis routes of *p*-bromostyrylphenanthroimidazoles, namely, 2-(4-bromostyryl)-1-(naphthalen-1-yl)-1*H*-phenanthro[9,10-*d*]imidazole (NSPI), 2-(4-bromostyryl)-1-(2-methylnaphthalen-1-yl)-1*H*-phenanthro[9,10-*d*]imidazole (MNSPI) and 4-(2-(4-bromostyryl)-1*H*-phenanthro[9,10-*d*]imidazol-1-yl)naphthalene-1-carbonitrile (SPNCN), along with donor–spacer–acceptor derivatives, namely, NSPI-TPA, MNSPI-TPA, SPNCN-TPA and SPNCN-Cz, are displayed in Scheme 1. Phenanthrene-9,10-dione (1 mmol), 4-bromo-1-cinnamaldehyde (1 mmol), 1-naphthylamine (NSPI)/2-methylnaphthalen-1-amine (MNSPI)/4-aminonaphthalene-1-carbonitrile (SPNCN) (1 mmol) and ammonium acetate (1 mmol) were refluxed in 25 mL acetic acid. The crude product was column chromatographed (hexane : ethylacetate) and the pure sample was used for synthesizing the blue emissive materials.

#### 2.1.1 4′-(1-(Naphthalen-1-yl)-1*H*-phenanthro[9,10-*d*]imidazol-2-yl)-*N*,*N*-diphenyl-(2-[1,1′-biphenyl]vinyl)-4-amine (NSPI-TPA)

Mixture of 2-(4-bromostyryl)-1-(naphthalen-1-yl)-1*H*-phenanthro[9,10-*d*]imidazole (NSPI) (4.5 mmol), 4-(diphenylamino)phenylboronic acid (7.5 mmol), Pd(PPh_3_)_4_ (0.25 mmol) and aqueous Na_2_CO_3_ (15 mL) in toluene : ethanol (20 : 15 mL) was refluxed (N_2_ atmosphere) for 18 h. The reaction mixture was extracted with dichloromethane and then, the solvent was distilled. The purified NSPI-TPA was used for further analysis. Yield 68%. Anal. calcd: C_51_H_35_N_3_: C, 88.79; H, 5.11; N, 6.09. Found: C, 88.72; H, 5.06; N, 6.01. 400 MHz ^1^H NMR (CDCl_3_): *δ* 6.58–6.61 (m, 6H), 6.64 (d, *J* = 8.2 Hz, 2H), 6.98 (d, *J* = 16.4 Hz, 2H), 7.14 (t, 4H), 7.31–7.38 (m, 10H), 7.7 (d, *J* = 8.4 Hz, 3H), 7.83–7.89 (m, 4H), 8.12 (d, *J* = 8.3 Hz, 2H), 8.93 (d, *J* = 8.6 Hz, 2H) (Fig. S1†). 400 MHz ^13^C NMR (CDCl_3_): *δ* 112.7, 122.32, 122.61, 124.21, 126.52, 126.84, 127.75, 127.94, 129.64, 130.68, 130.80, 131.47, 132.35, 133.58, 134.26, 134.68, 135.85, 139.56, 141.11, 141.62 (Fig. S2†). MALDI TOF MS: *m*/*z*. 689.21 [M^+^] (Fig. S9†). Calcd: 689.28.

#### 2.1.2 4′-(1-(2-Methylnaphthalen-1-yl)-1*H*-phenanthro[9,10-*d*]imidazol-2-yl)-*N*,*N*-diphenyl-(2-[1,1′-biphenyl]vinyl)-4-amine (MNSPI-TPA)

A mixture of 2-(4-bromostyryl)-1-(2-methylnaphthalen-1-yl)-1*H*-phenanthro[9,10-*d*]imidazole (MNSPI) (4.5 mmol), 4-(diphenylamino)phenylboronic acid (7.5 mmol), Pd(PPh_3_)_4_ (0.25 mmol) and aqueous Na_2_CO_3_ (15 mL) in toluene : ethanol (20 : 15 mL) was refluxed for 24 h and extracted with dichloromethane. Yield 58%. Anal. calcd: C_52_H_37_N_3_: C, 88.73; H, 5.30; N, 5.97. Found: C, 88.68; H, 5.25; N, 5.91. 400 MHz ^1^H NMR (CDCl_3_): *δ* 2.45 (s, 3H), 6.46–6.54 (m, 6H), 6.66 (d, *J* = 8.1 Hz, 2H), 6.91 (d, *J* = 16.3 Hz, 2H), 7.02 (t, 4H), 7.12–7.45 (m, 9H), 7.51–7.62 (m, 3H), 7.81–7.85 (m, 4H), 8.11 (d, *J* = 8.4 Hz, 2H), 8.91 (d, *J* = 8.2 Hz, 2H) (Fig. S3†). 400 MHz ^13^C NMR (CDCl_3_): *δ* 15.51, 112.70, 122.31, 123.15, 125.25, 126.36, 126.69, 126.81, 127.54, 127.72, 127.84, 128.41, 129.86, 131.44, 133.57, 134.82, 135.66, 139.80, 141.15, 147.75 (Fig. S4†). MALDI TOF MS: *m*/*z*. 703.26 [M^+^] (Fig. S9†). Calcd: 703.30.

#### 2.1.3 4-(2-(4′-(Diphenylamino)-(2-[1,1′-biphenyl]vinyl)-4-yl)-1*H*-phenanthro[9,10-*d*]imidazol-1-yl)-1-naphthonitrile (SPNCN-TPA)

A mixture of 4-(2-(4-bromostyryl)-1*H*-phenanthro[9,10-*d*]imidazol-1-yl)naphthalene-1-carbonitrile (SPNCN) (4.5 mmol), 4-(diphenylamino)phenylboronic acid (7.5 mmol), Pd(PPh_3_)_4_ (0.25 mmol) and aqueous Na_2_CO_3_ (15 mL) in toluene : ethanol (20 : 15 mL) was refluxed under N_2_ stream. Yield 60%. Anal. calcd: C_52_H_34_N_4_: C, 87.37; H, 4.79; N, 7.84. Found: C, 87.31; H, 4.72; N, 7.78. 400 MHz ^1^H NMR (CDCl_3_): *δ* 6.59–6.61 (m, 6H), 6.99 (d, *J* = 16 Hz, 2H), 7.15 (t, 4H), 7.25 (d, *J* = 8.2 Hz, 2H), 7.36–7.45 (m, 7H), 7.56–7.88 (m, 9H), 8.12 (d, *J* = 8.4 Hz, 2H), 8.93 (d, *J* = 8.5 Hz, 2H) (Fig. S5†). 400 MHz ^13^C NMR (CDCl_3_): *δ* 109.40, 110.61, 115.88, 121.65, 122.51, 123.49, 126.64, 126.81, 128.52, 128.64, 129.74, 131.87, 132.85, 133.58, 134.56, 135.18, 139.55 (Fig. S6†). MALDI TOF MS: *m*/*z*. 714.21 [M^+^] (Fig. S9†). Calcd: 714.28.

#### 2.1.4 4-(2-(4-(9*H*-Carbazol-9-yl)styryl)-1*H*-phenanthro[9,10-*d*]imidazol-1-yl)naphthalene-1-carbonitrile (SPNCN-Cz)

A mixture of 4-(2-styryl-1*H*-phenanthro[9,10-*d*]imidazol-1-yl)naphthalene-1-carbonitrile (SPNCN) (4.5 mmol), 9*H*-carbazole (7.5 mmol), (0.45 g, 1.0 mmol), CuI (10.0 mg, 0.05 mmol), 18-crown-6 (13.2 mg, 0.05 mmol) and K_2_CO_3_ (0.83 g, 6.0 mmol) in tetrahydro-1,3-dimethylpyrimidin-2(1*H*)-one (2.0 mL) was refluxed in ethanol (20 mL) under nitrogen atmosphere. Yield 61%. Anal. calcd: C_46_H_28_N_4_: C, 86.77; H, 4.43; N, 8.80. Found: C, 86.72; H, 4.38; N, 8.73. 400 MHz ^1^H NMR (CDCl_3_): *δ* 6.99 (d, *J* = 16.1 Hz, 2H), 7.0–7.08 (m, 4H), 7.22–7.31 (m, 4H), 7.45–7.66 (m, 7H), 7.80–7.89 (m, 6H), 8.12 (d, *J* = 8.4 Hz, 3H), 8.93 (d, *J* = 8.0 Hz, 2H) (Fig. S7†). 400 MHz ^13^C NMR (CDCl_3_): *δ* 109.50, 111.61, 112.81, 115.87, 119.12, 120.13, 121.01, 121.59, 121.74, 122.24, 122.48, 123.81, 126.54, 126.67, 127.56, 128.61, 131.51, 132.30, 132.75, 133.46, 136.55, 139.74, 14.35, 141.56 (Fig. S8†). MALDI TOF MS: *m*/*z*. 636.15 [M^+^] (Fig. S9†). Calcd: 636.23.

### 2.2 Measurements and general methods

Reagents and solvents were purchased from commercial sources. ^1^H and ^13^C NMR spectra were recorded on a Bruker (400 MHz) spectrometer and an Agilent instrument (LCMS VL SD) was employed to record the mass spectra. UV-vis absorption was measured using a Perkin-Elmer Lambda 35 (solution) and Lambda 35 spectrophotometer with an integrated sphere (RSA-PE-20) (film). Photoluminescence (PL) spectra were recorded on a PerkinElmer LS55 fluorescence spectrometer. Thermogravimetric analysis (TGA) and differential scanning calorimetry (DSC) were performed using a PerkinElmer thermal analysis system and NETZSCH-DSC-204, respectively. Decay analysis was conducted using a nanosecond time correlated single photon counting (TCSPC) spectrometer (Horiba Fluorocube-01-NL lifetime system) with nanoLED excitation source and TBX-PS detector; DAS6 software was used to fit the decay curve and *χ*^2^ values lie between 0.8 and 1.2. The absolute photoluminescence quantum yield (PLQY) was determined using a fluorescence spectrometer (Model-F7100). Cyclic voltammetry was performed using Potentiostat CHI 630A electrochemical analyzer (platinum electrode and platinum wire as the working electrode and counter electrode, respectively; Ag/Ag^+^ as the reference electrode, with 100 mV s^−1^ scan). Ferrocene was used as the internal standard, with the highest occupied molecular orbital energy of −4.80 eV, and 0.1 M tetrabutylammoniumperchlorate in CH_2_Cl_2_ was used as the supporting electrolyte. The HOMO energies (*E*_HOMO_) were calculated using oxidation potential [*E*_HOMO_ = −(*E*_ox_ + 4.8 eV)] and the LUMO energies (*E*_LUMO_) were calculated by subtracting the optical band gap from the HOMO energy [*E*_LUMO_ = *E*_HOMO_ − 1239/*λ*_onset_]. For theoretical calculations, ground state (DFT)/excited state (TD-DFT) geometrical properties were optimized by employing Gaussian 09 program.^47^ Multifunctional wavefunction analyzer (Multiwfn)^29^ was used to determine the nature of electronic transitions of excited states and natural transition orbitals (NTOs).

### 2.3 Fabrication of devices

Devices with structure of ITO/NPB (70 nm)/NSPI-TPA or MNSPI-TPA or SPNCN-TPA or SPNCN-Cz (100 nm)/TPBI (20 nm)/LiF (0.5 nm)/Al (120 nm) were fabricated on pre-cleaned ITO-coated glass substrates with 20 Ω sq^−1^ sheet resistance. Current density–voltage characteristics were recorded on a Keithley 2400 power source. EL spectra were recorded on a USB-650-VIS-NIR spectrometer (Ocean Optics, Inc, USA).

## 3 Results and discussion

### 3.1 Synthesis, thermal and photophysical properties

The synthesis routes of *p*-bromostyrylphenanthrimidazoles (NSPI, MNSPI and SPNCN) and donor–spacer–acceptor derivatives (NSPI-TPA, MNSPI-TPA, SPNCN-TPA and SPNCN-Cz) are shown in Scheme 1. The blue emitters were synthesized *via* Suzuki coupling reactions^48^ of *p*-bromostyrylphenanthrimidazoles with 4-diphenylaminophenylboronic acid (NSPI-TPA, MNSPI-TPA and SPNCN-TPA)/9*H*-carbazole (SPNCN-Cz) with yields of 68, 58, 60 and 61% of NSPI-TPA, MNSPI-TPA, SPNCN-TPA and SPNCN-Cz, respectively. All these as-synthesized emitters were confirmed *via*^1^H and ^13^C NMR spectroscopy, elemental analysis and high-resolution mass spectrometry. The two new efficient cyano-substituted blue emissive materials, SPNCN-TPA and SPNCN-Cz, consist of triphenylamine-styryl-naphthalenecarbonitrilephenathrimidazole (TPA-strong donor) and carbazole-styryl-naphthalenecarbonitrilephenathrimidazole (Cz-weak donor) backbones and the orthogonal naphthonitrile acceptor. In NSPI-TPA, MNSPI-TPA and SPNCN-TPA, the styryl ring is inserted between triphenylamine and naphthonitrilephenanthrimidazole to extend the π-conjugation (increase the LE component), which results in the enhancement of PL efficiency (*η*_PL_) while simultaneously maintaining the strength of both donor (LE component) and acceptor (CT component). However, carbazole in SPNCN-Cz induces higher LE magnitude than SPNCN-TPA. To improve photoluminescence efficiency (*η*_PL_), the strong donor TPA moiety in SPNCN-TPA is replaced with weaker donor carbazole to decrease the CT component and increase the LE component in the emissive state (S_1_ HLCT) of SPNCN-Cz.

**Fig. 1:**
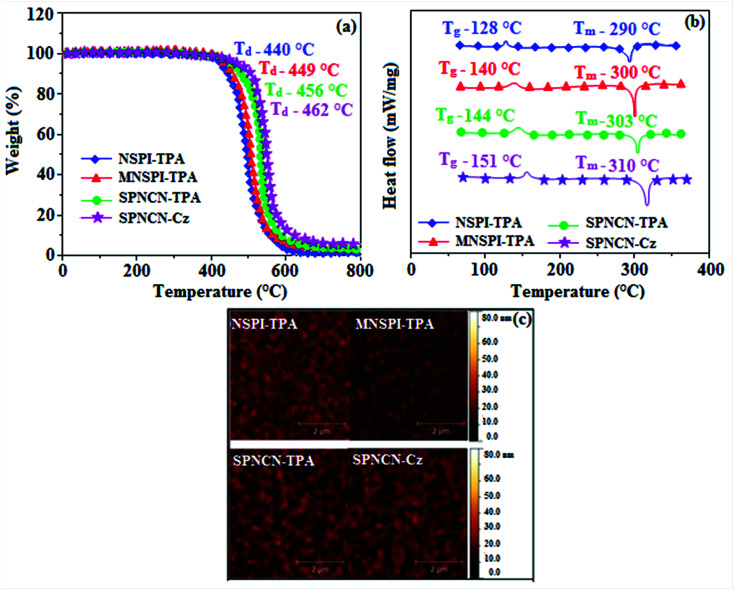
(a) TGA, (b) DSC graphs, and (c) AFM images of NSPI-TPA, MNSPI-TPA, SPNCN-TPA and SPNCN-Cz.

**Fig. 2:**
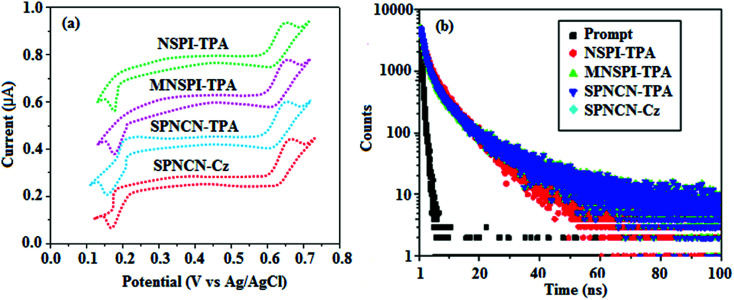
(a) Cyclic voltammogram and (b) lifetime spectra of NSPI-TPA, MNSPI-TPA, SPNCN-TPA and SPNCN-Cz.

**Fig. 3:**
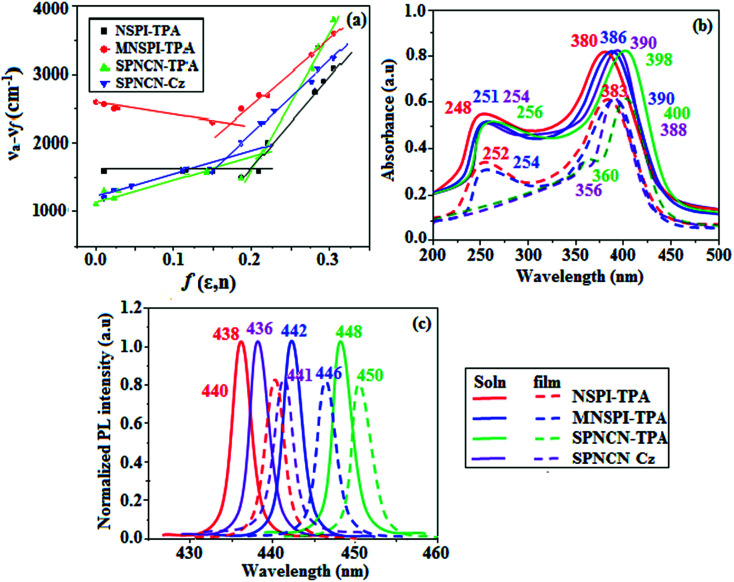
(a) Lippert–Mataga plots, (b) normalized absorption spectra, and (c) emission spectra of NSPI-TPA, MNSPI-TPA, SPNCN-TPA and SPNCN-Cz.

**Fig. 4:**
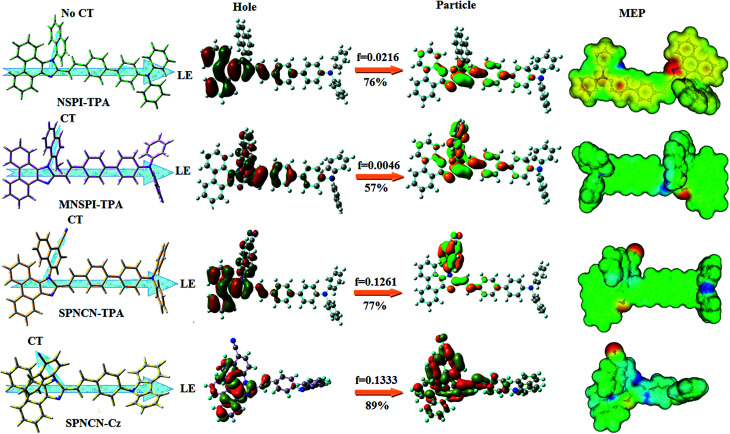
HOMO, LUMO contour maps and molecular electrostatic potential (ESP) surface along with CT/LE direction in NSPI-TPA, MNSPI-TPA, SPNCN-TPA and SPNCN-Cz.

**Fig. 5:**
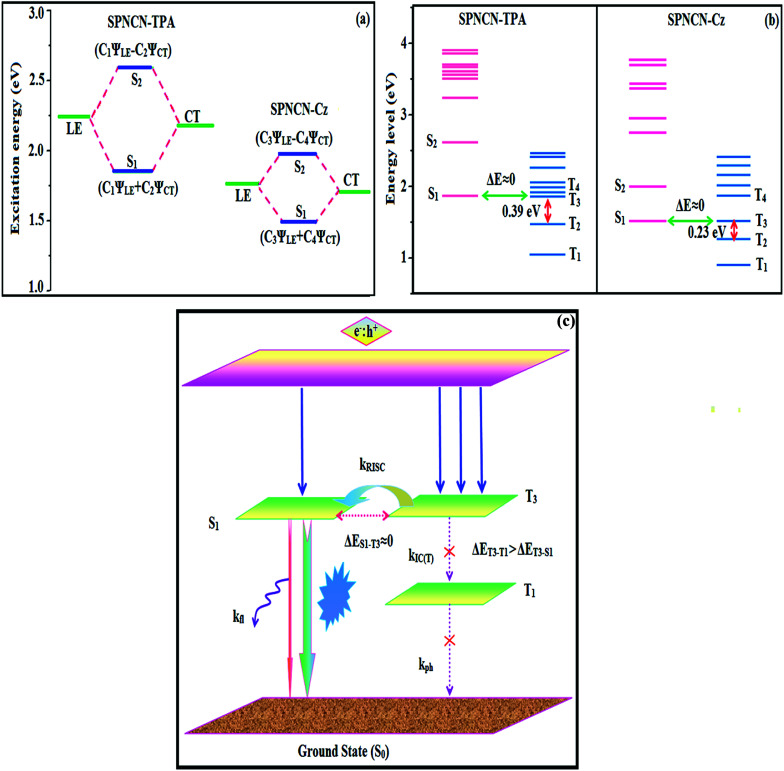
(a) Schematic of hybridization processes of LE and CT states of SPNCN-TPA and SPNCN-Cz; (b) energy levels of singlet (S) and triplet (T) states of SPNCN-TPA and SPNCN-Cz; (c) scheme of exciton decay process after hole and electron recombination in OLEDs of D–π–A molecules.

**Fig. 6:**
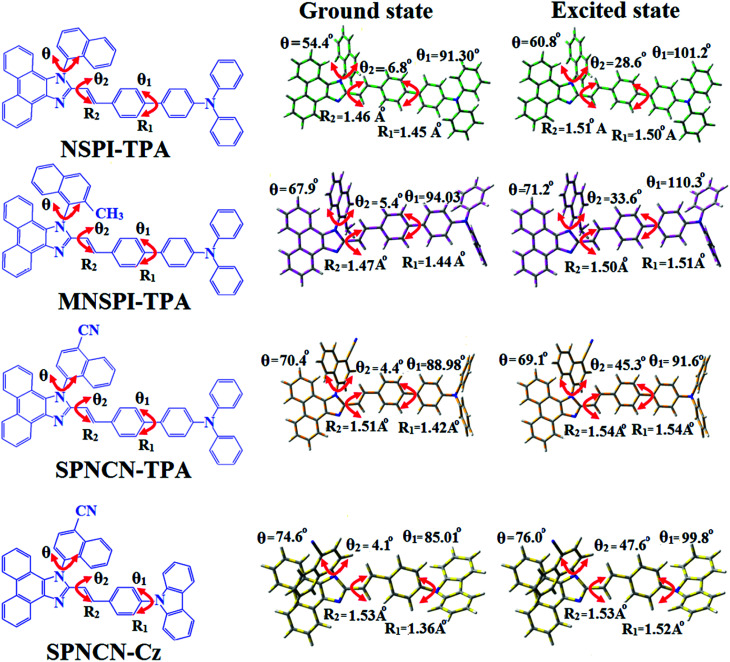
Ground state and excited state geometries with dihedral angles and bond lengths of NSPI-TPA, MNSPI-TPA, SPNCN-TPA and SPNCN-Cz.

**Fig. 7:**
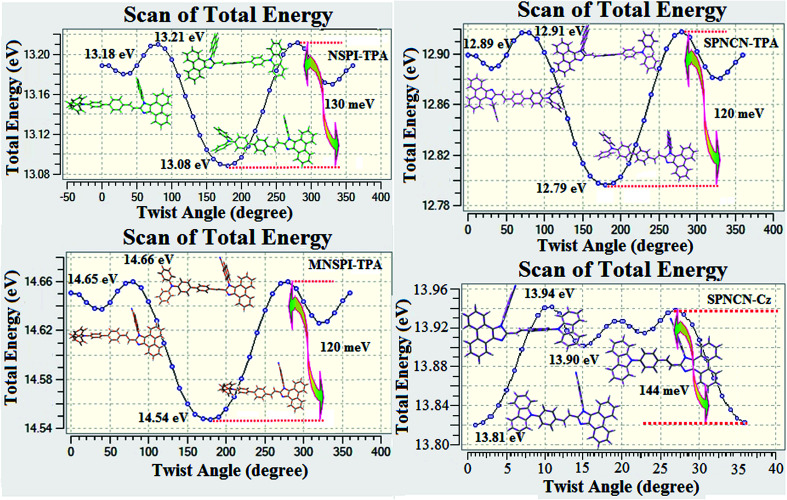
Potential energy curves of NSPI-TPA, MNSPI-TPA, SPNCN-TPA and SPNCN-Cz.

**Fig. 8:**
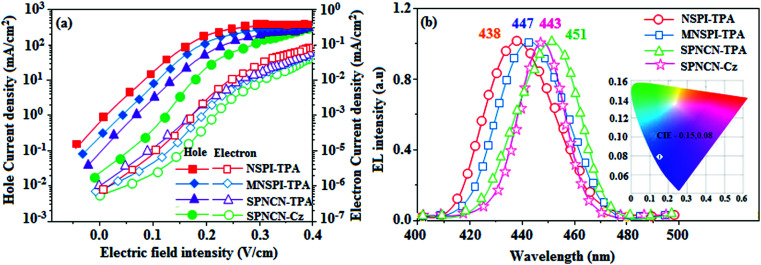
Hole-only and electron-only devices based on NSPI-TPA, MNSPI-TPA, SPNCN-TPA and SPNCN-Cz.

**Fig. 9:**
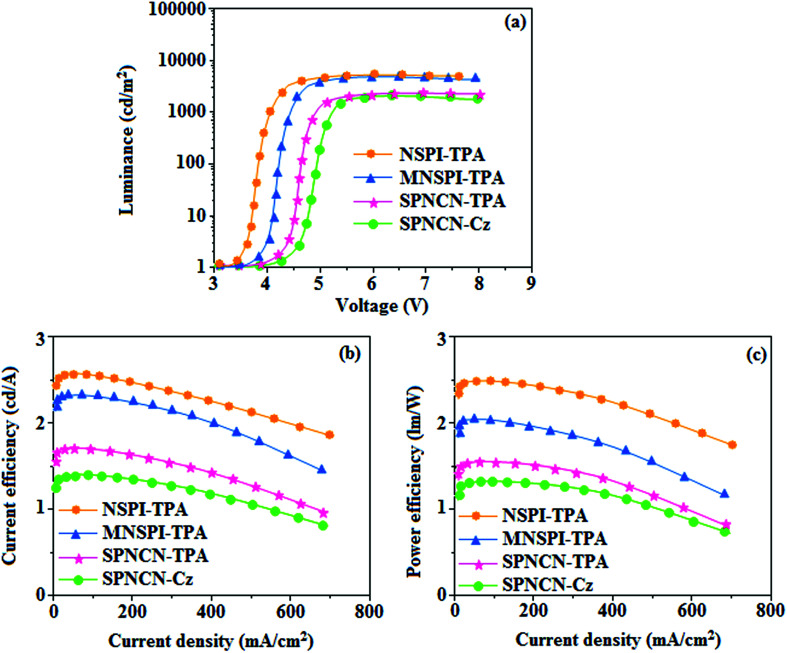
Electroluminescence performances of non-doped EL devices with NSPI-TPA, MNSPI-TPA, SPNCN-TPA and SPNCN-Cz as emitters: (a) luminance *versus* voltage, (b) current efficiency *versus* current density and (c) power efficiency *versus* current density.

The decomposition temperatures (*T*_d5_) of NSPI-TPA, MNSPI-TPA, SPNCN-TPA and SPNCN-Cz were measured to be 440, 449, 456 and 462 °C, respectively. The harvested high glass-transition temperature (*T*_g_) of 128, 140, 144 and 151 °C (Fig. 1) along with high melting temperatures (*T*_m_) of 290, 300, 303 and 310 °C are attributed to the non-coplanar geometry shown by bulky naphthonitrilephenanthrimidazole block along with triphenylamine/carbazole and styryl linker of rigid D–π–A molecules (Table 1). The thermal morphological stabilities of NSPI-TPA, MNSPI-TPA, SPNCN-TPA and SPNCN-Cz thin films were examined by atomic force microscopy (AFM) at room temperature and at 100 °C for 16 h. The root-mean-square roughness (RMS) of NSPI-TPA (0.25 nm), MNSPI-TPA (0.29 nm), SPNCN-TPA (0.35 nm) and SPNCN-Cz (0.39 nm) thin-film surfaces show that there are no substantial changes before and after annealing (100 °C, Fig. 1). The glass transition (*T*_g_) and decomposition temperatures (*T*_d5_) and the morphologies of NSPI-TPA, MNSPI-TPA, SPNCN-TPA and SPNCN-Cz thin films support the suitability of these materials for fabrication of OLEDs and hence, it is expected that the as-synthesized D–π–A materials will lower the turn on voltage in the device performances.^49,50^ The small HOMO energies (*E*_HOMO_) of −5.26 eV (NSPI-TPA), −5.23 eV (MNSPI-TPA), −5.20 eV (SPNCN-TPA) and −5.21 eV (SPNCN-Cz) were estimated from their respective oxidation onset potentials [0.46 V (NSPI-TPA), 0.43 V (MNSPI-TPA), 0.40 V (SPNCN-TPA) and 0.41 V (SPNCN-Cz)], supporting the hole-injection property of D–π–A materials (Fig. 2, Table 1). Since the LUMO energies (*E*_LUMO_) of −2.04 eV (NSPI-TPA), −2.06 eV (MNSPI-TPA), −2.02 eV (SPNCN-TPA) and 2.15 eV (SPNCN-Cz) are close to that of 1,3,5-tris(*N*-phenylimidazol-2-yl)benzene (−2.40 eV), these materials also possess electron-injection abilities.^51^ Furthermore, the frontier molecular orbital (HOMO–LUMO) analysis confirms the carrier injection abilities and hence, these materials can be employed as potential emitters in OLEDs.^52,53^ The calculated high fluorescence quantum yields, which are 0.58/0.46 (NPS-TPA), 0.69/0.56 (MNPS-TPA), 0.48/0.42 (SPNCN-TPA) and 0.52/0.49 (SPNCN-Cz), in both solution and film support the co-emission phenomenon from LE and CT of emissive materials. The radiative (*k*_r_) and non-radiative (*k*_nr_) transition rates of SPNCN-TPA and SPNCN-Cz were calculated from lifetime (*τ*) and quantum yield (*ϕ*) data. Compared with SPNCN-TPA, the increase in radiative rate (*k*_r_) and decrease in non-radiative rate (*k*_nr_) of SPNCN-Cz are also in agreement with the aim of our molecular design. Compared with parent compounds, the blue emitting NSPI-TPA, MNSPI-TPA, SPNCN-TPA and SPNCN-Cz materials show very strong absorption [solution (*ε*_max_)/film] at 380 (*ε*_max_ = 26 315 cm^−1^ M^−1^)/383 nm, 386 (*ε*_max_ = 25 906 cm^−1^ M^−1^)/390 nm, 398 (*ε*_max_ = 25 125 cm^−1^ M^−1^)/400 nm and 390 (*ε*_max_ = 25 641 cm^−1^ M^−1^)/388 nm (Fig. 3). The intramolecular charge transfer (ICT) from donor triphenylamine/carbazole to acceptor (naphthonitrilephenanthrimidazole) is likely to be the cause for strong absorption, and the absorption at around 248 nm is attributed to π–π* transition.^54^ The intramolecular charge transfer (ICT) was also confirmed by the MEP diagram (Fig. 4). Compared with solution of the materials, negligible absorption shifts in their corresponding films reveal that suppressed π–π* stacking exists in the solid state.^55^ The observed larger red shift further supports the charge-transfer (CT) in the twisted geometry of NSPI-TPA, MNSPI-TPA, SPNCN-TPA and SPNCN-Cz. Compared with SPNCN-TPA, SPNCN-Cz exhibits higher blue shift for both absorption and emission, which is attributed to the poor electron donor ability of Cz relative to TPA. The increase in LE composition with decrease in CT in the S_1_-emissive HLCT state is likely to be the reason for this blue shift. The full width at half maximum in the absorption spectrum of SPNCN-Cz (40 nm) is narrowed relative to that of SPNCN-TPA (58 nm, Fig. 3). This observation indicated that the decrease in CT component of SPNCN-Cz in the S_1_ state is in good agreement with the NTO description for S_0_ → S_1_ transition. However, the absorption peaks of both SPNCN-TPA and SPNCN-Cz are narrower than those of the cyano-free parent compounds, indicating more LE character. The emission peaks of SPNCN-TPA and SPNCN-Cz show blue shift relative to those of their parent compounds, which is in contrast to the general observation, *i.e.*, extension of π-conjugation leads to a red-shifted emission.^56^ The enhanced LE component is equivalent to the suppressed CT component in the emissive states of SPNCN-TPA and SPNCN-Cz, resulting in blue shift. The increase in LE composition is expected to result in a red-shifted PL spectrum, whereas the suppressed CT results in a blue-shifted PL spectrum. From the experimental observation, it is known that the latter factor is more dominant than the former. In addition, there is an overlap between UV and PL spectra of both SPNCN-TPA and SPNCN-Cz because of the enhanced LE character in SPNCN-TPA and SPNCN-Cz than that in their respective parent compounds. SPNCN-Cz exhibits solvatochromic red shift (45 nm), which is smaller than that of SPNCN-TPA (75 nm) (Fig. S10, Table S1 and S2†). Similarly, small absorption shifts of 22 nm and 32 nm were observed for SPNCN-Cz and SPNCN-TPA, respectively (Fig. S11, Tables S1 and S2†). These solvatochromic shifts confirmed that the low-lying S_1_-excited states of SPNCN-Cz and SPNCN-TPA must possess CT character.^57–59^ The % CT character of the S_1_ state of SPNCN-Cz is lower than that of the S_1_ state of SPNCN-TPA, whereas the % LE character of the S_1_ state of SPNCN-Cz is higher than that of the S_1_ state of SPNCN-TPA (Table 2). In hexane, both SPNCN-Cz and SPNCN-TPA show LE-like character because of vibronic PL spectrum. Compared with the broad and smooth PL spectrum of cyano-free compounds, there is higher % LE in the S_1_ state of both SPNCN-Cz and SPNCN-TPA. The full width at half maximum (FWHM) of SPNCN-Cz (40 nm) and SPNCN-TPA (58 nm) indicates that these compounds possess higher PL efficiency due to higher % LE in the S_1_ emissive state. The solvatochromic effect using Lippert–Mataga plot is displayed in Fig. 3 (Tables S1 and S2†). When the solvent polarity increased, the blue emitters exhibited larger red shift, which supports charge transfer (CT) in these molecules.^57^ From the plot of Stokes shift against solvent polarity function, ground state dipole moment (*μ*_g_) can be calculated:
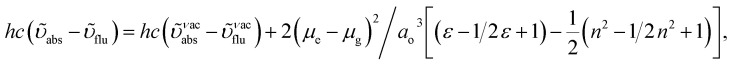
where *μ*_g_ and *μ*_e_ are the ground state and excited state dipole moments, *υ̃*_abs_ and *υ̃*^νac^_abs_ are the solvent-equilibrated absorption maximum and that extrapolated to gas phase, respectively, *υ̃*_flu_ and *υ̃*^νac^_flu_ are the solvent equilibrated fluorescence maximum and that extrapolated to gas-phase, respectively, *a*_o_ is the Onsager cavity and *ε* and *n* are the solvent dielectric constant and refractive index, respectively. The non-linear correlation of Stokes shift *vs.* solvent polarity function reveals that there is a transformation of the fitted line between ethyl ether and methylene chloride. This non-linear correlation supports the presence of both locally exited state (LE) and charge transfer exited state (CT). Ground state dipole (*μ*_g_) of blue-emitting materials NSPI-TPA, MNSPI-TPA, SPNCN-TPA and SPNCN-Cz could be estimated from density functional theory (DFT) calculations as 2.98, 3.39, 5.94 and 7.02 *D*, respectively, and the corresponding calculated *μ*_e_ values in high polarity solvents are 22.8, 23.6, 25.8 and 30.8 *D*. The small *μ*_g_ estimated from the slope is 2.98, 3.39, 5.94 and 7.02 *D* for NSPI-TPA, MNSPI-TPA, SPNCN-TPA and SPNCN-Cz, respectively, which is attributed to local exciton (LE) transition.^57^ The large calculated *μ*_e_ values in high polarity solvents (22.8, 23.6, 25.8 and 30.8 *D*) are close to the *μ*_e_ of the charge-transfer molecule 4-(*N*,*N*-dimethylamino)-benzonitrile (23.0 *D*).^59^ All these results show that CT dominates in high polarity medium, whereas LE dominates in low polarity medium and there is mixed contribution of LE and CT in medium polarity solvents (ethyl ether and methylene chloride).^31^ The oscillator strengths of NSPI-TPA, MNSPI-TPA, SPNCN-TPA and SPNCN-Cz are displayed in Tables 3 and 4. The oscillator strength of S_1_ state of SPNCN-Cz (0.2554, Table 4) is higher than that of SPNCN-TPA (0.1261, Table 4), which results in the higher PL efficiency (*η*_PL_) of SPNCN-Cz. Molecular modification from TPA to Cz caused an increase in % LE in the S_1_ emissive state and enhanced the *η*_PL_ of SPNCN-Cz. In order to supplement experimental results, theoretical calculations (natural transition orbital analysis) were performed to describe the excited state characteristics of SPNCN-TPA and SPNCN-Cz materials (Fig. 4). In SPNCN-TPA and SPNCN-Cz, holes are located over the horizontal backbone, whereas particles are located on vertical naphthonitrile in the S_1_ state. CT transition is maintained from the horizontal backbone to vertical naphthonitrile in the S_1_ states of SPNCN-TPA and SPNCN-Cz. The overlap density between a hole and a particle is expanded due to spacer styryl moiety, indicating an enhanced % LE in the S_1_ state (Fig. S12 and S13†). The NTOs of S_1_ and S_2_ excited states of SPNCN-TPA and SPNCN-Cz exhibit a hybrid splitting state character that derives from the interstate coupling of LE and CT levels (Tables 3, 4, Fig. S14 and S15†), *i.e.*, formation of HLCT state. The wave function symmetry of a particle on naphthonitrile is opposite between S_1_ and S_2_ states, indicating interstate hybridization coupling *Ψ*_S_1_/S_2__ = *c*_LE_*Ψ*_LE_ ± *c*_CT_*Ψ*_CT_. Similar hole–electron wave functions between S_1_ and S_2_ are observed in both SPNCN-TPA and SPNCN-Cz, indicating a quasi-equivalent hybridization between LE and CT states as a result of the almost isoenergies of initial LE and CT states (Fig. 5). Therefore, the degree of hybridization between LE and CT states is dependent not only on the initial *E*_LE_–*E*_CT_ energy gap but also on their interstate coupling strength.^60^ Compared with non-equivalent hybridization, quasi-equivalent hybridization is expected to achieve the combination of high *η*_PL_ and high *η*_s_ to maximize the EL efficiency of fluorescent OLED materials due to the more balanced LE and CT components in HLCT states of SPNCN-TPA and SPNCN-Cz. The formation of the HLCT state can be analysed through the excitation energies of LE and CT states (Tables 3 and 4). In SPNCN-Cz and SPNCN-TPA, the LE state is stabilized as compared with the CT state and energy gap (*E*_S_2__–*E*_S_1__) is small when compared with their cyano-free parent compounds, resulting in quasi-hybridization. In the case of SPNCN-Cz, the energy gap (*E*_S_2__–*E*_S_1__) is reduced, resulting in effective hybridization and improved OLED efficiency. The overlap between the hole and the particle is also displayed in Fig. S12† (SPNCN-TPA) and Fig. S13† (SPNCN-Cz). Composition of HLCT can be determined from the wave function of electron–hole pair transition density matrix (TDM) plotted in a two-dimensional color-filled map (Fig. S16 and S17†). The axes represent the atom in a molecule, which is proportional to the probability of finding an electron and a hole in an atomic orbital located on a non-hydrogen atom. The diagonal represents the LE component localized on the main backbone, while the off–diagonal region shows the CT component. The qualitatively calculated percentages of LE and CT in S_1_–S_10_ and T_1_–T_10_ states are displayed in Table 2. This finding also supports that HLCT state contributes to hybridization, in addition to the LE and CT states. Upon excitation, an electron is transferred from a donor and localized on an acceptor. Depending upon intramolecular geometrical and electronic coupling, the transferred electron is delocalized from the region of nearby the donor molecule to the vicinity of the acceptor. This effect can be qualitatively studied by analyzing the electron density distribution at the ground and excited states. Computed electron–hole properties, distance between hole and electron, transition density, *H* and *t* indexes and RMSD of electrons and holes of SPNCN-Cz and SPNCN-TPA are displayed in Table S3–S8.† The integral value of a hole and an electron of SPICN-Cz is less than that in SPICN-TPA with transition density. The integral overlap of hole–electron distribution (*S*) is a measure of spatial separation of holes and electrons. The integral overlap (*S*) of holes and electrons and the distance (*D*) between centroids of holes and electrons evidence the existence of LE and CT states (Tables S5 and S7†). When compared with parent compounds, SPNCN-TPA and SPNCN-Cz have small *D* and high *S* values. However, small *D* and high *S* of SPNCN-TPA indicate that charge transfer (CT) is higher in percentage for the SPNCN-TPA isomer. The variation in dipole moment with respect to S_0_ state was also outputted, which was directly evaluated based on the position of centroids of holes and electrons. RMSD of holes or electrons characterizes their distribution breadth (Tables S6–S8†). RMSDs of both electron and hole in SPNCN-Cz are higher in the *X* direction, indicating that electron and hole distributions are much broader in the *X* direction, whereas RMSD of the electron of SPNCN-TPA is smaller and that of the hole is higher than those of SPNCN-Cz. The *H* index (half sum of the axis of anisotropic density variation distribution) measures the spread of positive and negative regions related to CT. The CT index, *i.e.*, *t* index difference between *D*_CT_ and *H* index, is another measure of the separation of hole–electron (eqn (S15) and (S16)†). For both SPNCN-Cz and SPNCN-TPA, the non-zero *t* is negative in all directions. The overlap of the hole and electron is very severe (Table S9†) and the eigenvalue is greater than 0.96, supporting the hybridization and described in terms of dominant excitation pair in terms of 94% of transition. This is further evidenced by Δ*r* index (Tables S3 and S4†). The Δ*r* index (eqn (S1)†) is the average hole (h^+^)–electron (e^−^) distance (*d*_h^+^–e^−^_) upon excitation, which reveals the nature of the excitation, *viz.*, LE or CT. Valence excitation (LE) is related to short distances (*d*_h^+^–e^−^_), while larger distances (*d*_h^+^–e^−^_) are related to CT excitation. The triplet exciton is transformed to the singlet excitons in SPNCN-TPA and SPNCN-Cz *via* RISC process with high energy excited state (hot CT channel),^61,62^ which is beneficial for triplet exciton conversion in electroluminescence processes without any delayed fluorescence. CT excitons are formed with weak binding energy (*E*_b_) in higher excited states.^63^ As a result, the exciton utilization can be harvested in SPNCN-TPA and SPNCN-Cz, similar to that observed in phosphorescent materials. The quasi-equivalent hybridized materials SPNCN-TPA and SPNCN-Cz exhibit excellent device performances due to fine modulation in the excited states. The enhanced LE component and hybridization between LE and CT components results in high *η*_PL_ and high *η*_s_. The coexisting LE/CT composition in SPNCN-TPA and SPNCN-Cz harvested high *η*_PL_ and high *η*_s_ and enhanced the OLEDs' performances (Table 1). Ground (S_0_) and excited (S_1_) geometries of NSPI-TPA, MNSPI-TPA, SPNCN-TPA and SPNCN-Cz were optimized by DFT/B3LYP/6-31G (d,p) and TD-DFT/B3LYP/6-31G (d,p). The optimized geometry shows styryl in NSPI-TPA, MNSPI-TPA, SPNCN-TPA and SPNCN-Cz and the naphthonitrilephenanthrimidazole ring adopts a coplanar configuration with dihedral angles (*θ*_2_) of 6.8, 5.4, 4.4 and 4.1°, respectively, leading to an extended molecule. However, the dihedral angles (*θ*_1_) between the phenyl moiety of TPA and styryl ring are 91.30, 94.03, 88.98 and 85.01°. The highly twisted dihedral angles are beneficial to adjusting the π-conjugation length between acceptor and donor groups. Naphthonitrile is twisted with respect to the phenanthrimidazole ring with dihedral angles (*θ*) of 54.4 (NSPI-TPA), 67.9 (MNSPI-TPA), 70.4 (MNPS-TPA) and 74.6° (SPNCN-Cz). The donor TPA and acceptor phenanthrimidazole lead to electrostatic interactions^52^ in these molecules. The dihedral angle (*θ*_3_) between phenyl of TPA and the styryl moiety is a key parameter: larger angle suppresses π–π stacking in film, resulting in the prevention of the self-quenching of fluorescence. The twist angle (*θ*_2_) of the emitters in excited state is increased to 28.6, 33.6, 45.3 and 47.6° compared with ground state twist angle *θ*_2_ and bond length (*R*_1_) is elongated S_1_ → S_0_ by 0.05, 0.07, 0.14 and 0.16 Å (Fig. 6). The smaller change in geometry (S_0_ to S_1_) decreases the non-radiative emission (*k*_nr_), which results in enhanced photoluminescence efficiency (*η*_PL_). The twisted naphthonitrilephenanthrimidazoles NSPI-TPA, MNSPI-TPA, SPNCN-TPA and SPNCN-Cz can effectively suppress molecular aggregation and the almost orthogonal dihedral angles (∼89.0°) between styryl and phenyl of TPA/Cz core can effectively minimize the intermolecular packing (Fig. 7) and can be used as hole-trapping sites, whereas the peripheral phenanthrimidazole core blocks electron-trapping sites. Thus, both carrier injection and transport ability are expected from these reported emitters. Furthermore, the relative carrier transport of the title materials was investigated by fabricating hole-only devices as well as electron-only devices (Fig. 8). The hole-only and electron-only devices with configurations of ITO/HATCN (10 nm)/NPB (70 nm)/NSPI-TPA or MNSPI-TPA or SPNCN-TPA or SPNCN-Cz (100 nm)/NPB (70 nm)/Al (120 nm) (hole-only device IV) and ITO/TPBi (10 nm)/NSPI-TPA or MNSPI-TPA or SPNCN-TPA or SPNCN-Cz (100 nm)/TPBi (10 nm)/LiF (1 nm)/Al (120 nm) (electron-only device V) were fabricated. For the hole-only device, NPB adjacent to Al was deposited (to block electrons), whereas for the electron-only device, TPBi was deposited close to the anode (to avoid hole injection).^64^ The title materials show bipolar transporting abilities: the carrier mobility of compounds SPNCN-Cz and SPNCN-TPA was more effective than that of NPS-TPA and MNPS-TPA because of the naphthonitrile fragment. Hence, the devices based on SPNCN-TPA and SPNCN-Cz achieved relatively lower turn-on voltages with higher device efficiencies. Generally, TADF materials exhibit flat decay curves due to the time-consuming TADF process for exciton conversion from triplet to singlet. SPNCN-TPA and SPNCN-Cz decay sharply (Fig. 2). Hence, radiative excitons in SPNCN-TPA and SPNCN-Cz are short-lived without TADF contribution. Exciton utilization efficiency (*η*_s_) in SPNCN-TPA and SPNCN-Cz follows neither TTA nor TADF mechanism.^65^ The non-doped EL devices were fabricated to investigate the relationship between excited state properties and EL performances of NSPI-TPA, MNSPI-TPA, SPNCN-TPA and SPNCN-Cz (Fig. 9, Table 1). The device with configuration of ITO/NPB (70 nm)/NSPI-TPA (100 nm) or MNSPI-TPA (100 nm) or SPNCN-TPA (100 nm) or SPNCN-Cz (100 nm)/TPBi (20 nm)/LiF (1 nm)/Al (120 nm) were fabricated. The devices based on SPNCN-TPA and SPNCN-Cz exhibit blue EL emission with CIE of (0.15, 0.08) for SPNCN-TPA and (0.15, 0.07) for SPNCN-Cz. Among strong donor compounds, the SPNCN-TPA-based device exhibited blue emission at 451 nm, maximum current efficiency of 2.32 cd A^−1^, power efficiency of 2.01 lm W^−1^ and external quantum efficiency of 3.02%. The non-doped device with SPNCN-Cz exhibited better electroluminescent performance than the SPNCN-TPA-based device: high external quantum efficiency of 3.15%, current efficiency of 2.56 cd A^−1^ and power efficiency of 2.45 lm W^−1^. The *η*_s_ of SPNCN-Cz and SPNCN-TPA was calculated to be 32.14 and 30.8%, respectively, which was superior to the 25% spin statistics limit. The increased *η*_s_ and *η*_IQE_ (15.75 for SPNCN-Cz and 15.10 for SPNCN-TPA) was due to the CT state contributed from the cyano group. The twisted geometry of emissive materials through introduction of a sterically hindered group enhanced the color purity. The fabricated devices also show excellent efficiency with no roll-off external quantum efficiency. Efficiencies of the as-fabricated current devices were compared with literature reported efficiencies^66–79^ and displayed in Table S10,† which shows that newly synthesized twisted D–π–A blue emitters are the best in view of efficiency (Table 5).

**Table 1:** Photophysical and thermal properties and device efficiencies of NSPI-TPA, MNSPI-TPA, SPNCN-TPA and SPNCN-Cz

Parameters	NSPI-TPA	MNSPI-TPA	SPNCN-TPA	SPNCN-Cz
**Photophysical & thermal properties**				
a *λ*_ab_ (nm) (sol/film)	248, 380/252, 383	251, 386/254, 390	256, 398/360, 400	254, 390/356, 388
a *λ*_em_ (nm) (sol/film)	438/440	442/446	448, 450	439/441
FWHM	75	69	58	40
b *T*_m_/*T*_g_/*T*_d5_ (°C)	290/128/440	300/140/449	303/144/456	310/151/462
c *ϕ* (sol/film)	0.58/0.46	0.69/0.56	0.48/0.42	0.52/0.49
*τ* (ns)	2.5	2.1	1.7	1.8
dHOMO/LUMO (eV)	−5.26/−2.04	−5.23/−2.06	−5.20/−2.02	−5.21/−2.15
*k*_r_ × 10^8^ (s^−1^)	2.3	3.2	2.8	2.9
*k*_nr_ × 10^8^ (s^−1^)	1.7	1.5	3.0	2.7
e *E*_g_ (eV)	3.22	3.17	3.18	2.86

**Device efficiency**				
g *η*_IQE_ (%)	10.1	11.8	15.1	15.75
h *η*_s_ (%)	20.6	21.1	30.8	32.14
*V*_on_ (V)	4.5	4.3	3.8	3.5
*L* (cd m^−2^)	2013	2158	4362	4826
f *η*_ex_ (%)	2.01	2.36	3.02	3.15
*η*_c_ (cd A^−1^)	1.38	1.68	2.32	2.56
*η*_p_ (lm W^−1^)	1.29	1.51	2.01	2.45
EL (nm)	438	447	451	443
CIE (*x*, *y*)	(0.15, 0.12)	(0.15, 0.10)	(0.15, 0.08)	(0.15, 0.07)

a Normalized absorption (*λ*_ab_) and emission (*λ*_em_) spectra of NSPI-TPA, MNSPI-TPA, SPNCN-TPA and SPNCN-Cz in CH_2_Cl_2_ (10–5 M)/film.

b *T*_g_/*T*_d_-glass transition temperature/thermal decomposition temperature at a weight percentage of 95%.

c *ϕ* (soln/film) – PL quantum yield was calculated in dichloromethane/solid state quantum yield was measured on the quartz plate using an integrating sphere.

d HOMO/LUMO – *E*_HOMO_ = (*E*_ox_ + 4.8 eV)/*E*_LUMO_ = *E*_HOMO_ − 1239/*λ*_onset_.

e *E*_g_ – energy gap (HOMO–LUMO).

f *η*_ex_ – external quantum efficiency; maximum internal quantum efficiency.

g *η*_IQE_ = *η*_ex_/*η*_out_, *η*_out_ light out coupling efficiency (−20%); excitation utilization efficiency.

h *η*_s_ = *η*_IQE_/*η*_PL_.

**Table 2:** Percentage of LE and CT in SPNCN-TPA and SPNCN-Cz

Percentage of transition	SPNCN-TPA		SPNCN-Cz	
	Singlet S_1_–S_10_	Triplet T_1_–T_10_	Singlet S_1_–S_10_	Triplet T_1_–T_10_
% LE	10	60	30	40
% CT	90	40	70	60

**Table 3:** Calculated energies (*E*, eV) and oscillator strengths (*f*) of S_0_–S_10_ transitions from NTO of NSPI-TPA and MNSPI-TPA

Transitions	NSPI-TPA			MNSPI-TPA		
	*E*	*f*	NTO	*E*	*f*	NTO
S_0_ → S_1_	1.06	0.0216	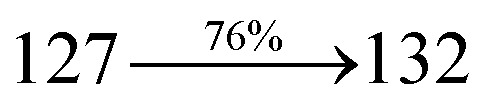	1.93	0.0187	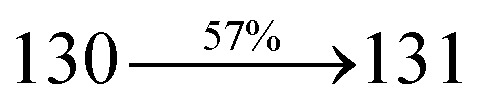
S_0_ → S_2_	1.43	0.0215	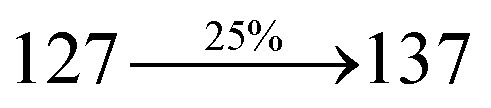	2.76	0.0850	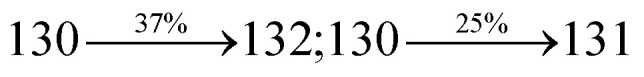
S_0_ → S_3_	1.65	0.0056	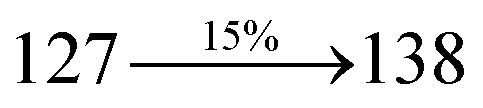	3.17	0.5536	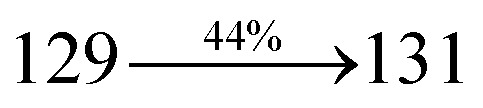
S_0_ → S_4_	1.81	0.0011	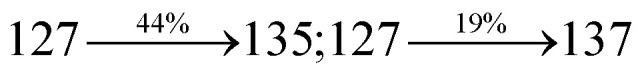	3.24	0.0370	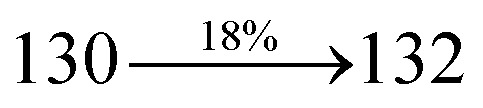
S_0_ → S_5_	2.90	0.0024	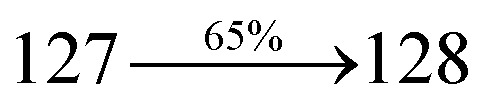	3.37	0.1357	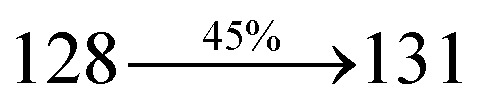
S_0_ → S_6_	3.05	0.1315	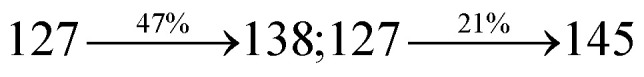	3.56	0.1629	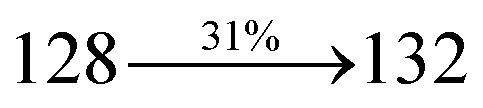
S_0_ → S_7_	3.24	0.5378	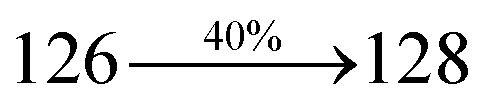	3.61	0.0669	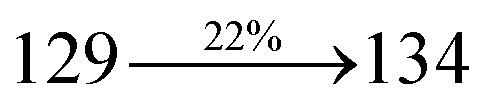
S_0_ → S_8_	3.27	0.0334	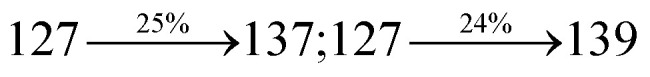	3.66	0.2855	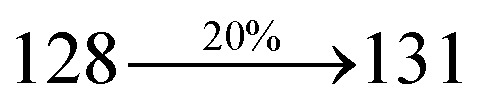
S_0_ → S_9_	3.32	0.0369	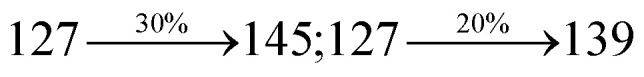	3.87	0.0723	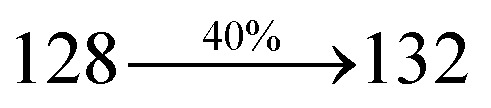
S_0_ → S_10_	3.40	0.5391	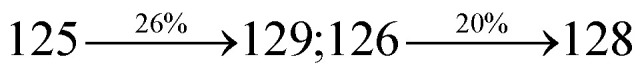	3.91	0.2707	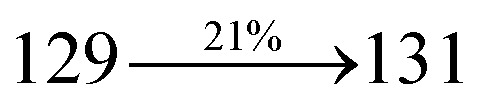

**Table 4:** Calculated energies (*E*, eV) and oscillator strengths (*f*) of S_0_–S_10_ transitions from NTO of SPNCN-TPA and SPNCN-Cz

Transitions	SPNCN-TPA			SPNCN-Cz		
	*E*	*f*	NTO	*E*	*f*	NTO
S_0_ → S_1_	2.57	0.1261	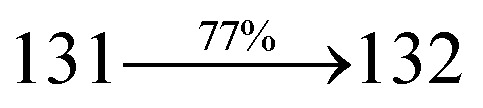	2.08	0.2554	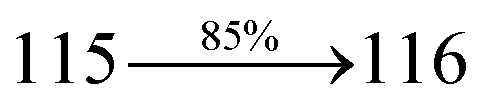
S_0_ → S_2_	3.23	0.2437	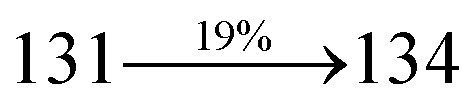	2.72	0.2762	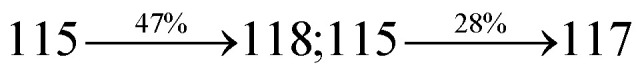
S_0_ → S_3_	3.23	0.5362	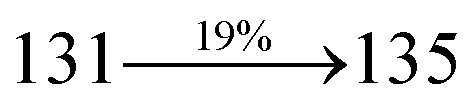	2.74	0.0865	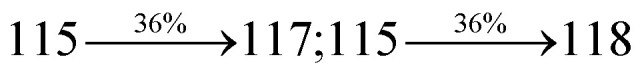
S_0_ → S_4_	3.51	0.0665	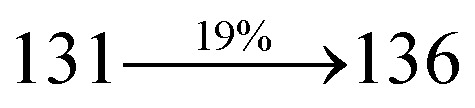	2.94	0.0637	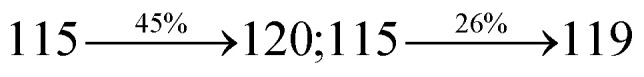
S_0_ → S_5_	3.58	0.1319	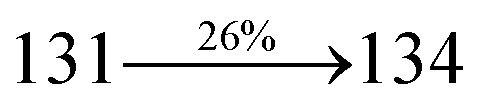	3.34	0.1165	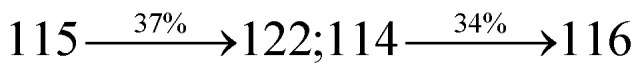
S_0_ → S_6_	3.66	0.0972	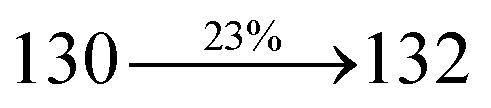	3.42	0.1741	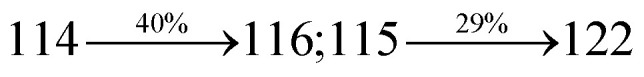
S_0_ → S_7_	3.86	0.2489	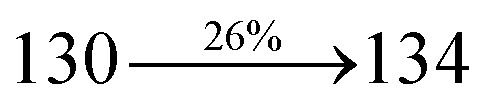	3.68	0.0986	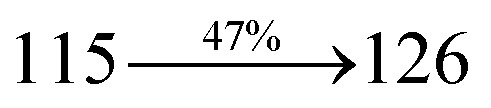
S_0_ → S_8_	3.87	0.4764	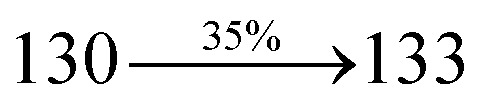	3.75	0.1179	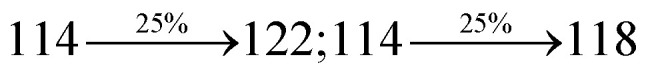
S_0_ → S_9_	3.98	0.1723	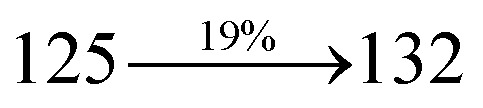	3.86	0.2818	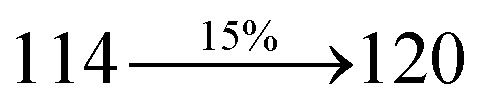
S_0_ → S_10_	4.05	0.2212	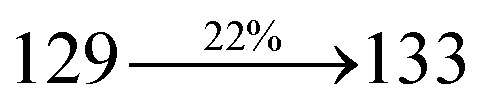	3.91	0.0054	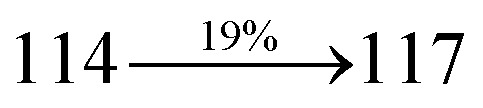

**Table 5:** Excitation energy (*E*, eV), excitation co-efficient (*ε*), overlap integral (Δ*r*, Å) and nature of transition of S_1_ → S_10_ states of NSPI-TPA and MNSPI-TPA

Excited states	NSPI-TPA			MNSPI-TPA		
	*E*	*ε*	Δ*r*	*E*	*ε*	Δ*r*
S_1_	1.0619	0.4317	1.4304	1.9290	0.4138	1.0838
S_2_	1.4258	0.4552	3.3486	2.7584	0.3950	1.6500
S_3_	1.6516	0.4627	3.6408	3.1700	0.3886	4.0225
S_4_	1.8057	0.4813	2.0202	3.2444	0.3897	3.0161
S_5_	2.8968	0.4279	5.3083	3.3693	0.3787	3.1412
S_6_	3.0497	0.4038	3.1612	3.5633	0.3749	3.6268
S_7_	3.2426	0.3771	3.0296	3.0096	0.4094	3.8344
S_8_	3.2672	0.4361	2.1519	3.6624	0.3527	2.9801
S_9_	3.3180	0.3911	2.7920	3.8681	0.3589	2.9531
S_10_	3.3992	0.3976	2.8895	3.9141	0.3501	3.8249

## 4 Conclusions

We have reported new deep-blue emitters using twisted donor–π–acceptor molecular design strategy. The photophysical and thermal stabilities and electrochemical properties of cyano-substituted blue fluorescent materials SPNCN-TPA and SPNCN-Cz can be modulated by the chemical modification of TPA moiety by a Cz fragment, which results in an HLCT emissive state with the increase in LE, decrease in CT and an increase in quantum efficiency. A fine modulation of the emissive state was performed between LE and CT composition to form a quasi-equivalent hybridized HLCT state in SPNCN-Cz and SPNCN-TPA, in which the LE component contributes high *η*_PL_, whereas the CT component generates high *η*_s_. SPNCN-Cz-based device shows external quantum efficiency of 3.28%, current efficiency of 2.90 cd A^−1^ and power efficiency of 2.26 lm W^−1^. The molecular design of the twisted conformation can be used to fabricate low cost fluorescent OLED materials using HLCT state principle.

## Conflicts of interest

There are no conflicts of interest.

## Supplementary Material

RA-008-C8RA07891B-s001
