# Supplementary material for: Hot exciton transition for organic light-emitting diodes: tailoring excited-state properties and electroluminescence performances of donor–spacer–acceptor molecules
Source: RSC Adv. 2018 Nov 6;8(65):37324–38. doi: 10.1039/c8ra07891b (PMC9088966; doi:10.1039/c8ra07891b)
Supplement: RA-008-C8RA07891B-s001 [file RA-008-C8RA07891B-s001.pdf]

Electronic Supplementary Material (ESI) for RSC Advances.
This journal is © The Royal Society of Chemistry 2018

Supporting information

Hot excition transition for organic light-emitting diodes: Tailoring excited-state properties and electroluminescence performances of donor-spacer-acceptor molecules

Jayaraman Jayabharathi*, Sekar Panimozhi, Venugopal Thanikachalam

Department of Chemistry, Annamalai University, Annamalainagar 608 002, Tamilnadu, India

* Tel: +91 9443940735; *E-mail address:* jtchalam2005@yahoo.co.in.

Contents

SI–I: Charge-Transfer intexes

SI–XVII: Figures

SI–X: Tables

SI–I: Charge–Transfer intexes

The hole–particle pair interactions have been related to the distance covered during the excitations one possible descriptor Δr intex could be used to calculate the average distance which is weighted in function of the excitation coefficients.

$$\Delta r = \frac{\Sigma_{ia} k_{ia}^2 \left| \langle \varphi_a | r | \varphi_a \rangle - \langle \varphi_i | r | \varphi_i \rangle \right|}{\Sigma_{ia} K_{ia}^2} \quad \text{................ (S1)}$$

where $\left| \langle \varphi_i | r | \varphi_i \rangle \right|$ is the norm of the orbital centroid [1–4]. Δr–index will be expressed in Å. The density variation associated to the electronic transition is given by

$$\Delta\rho(r) = \rho_{EX}(r) - \rho_{GS}(r) \quad \text{..................... (S2)}$$

where $\rho_{GS}(r)$ and $\rho_{EX}(r)$ are the electronic densities of to the ground and excited states, respectively. Two functions, $\rho_+(r)$ and $\rho_-(r)$, corresponds to the points in space where an increment or a depletion of the density upon absorption is produced and they can be defined as follows:

$$\rho_+(r) = \left\{ \begin{matrix} \Delta\rho(r) & if\ \Delta\rho(r) > 0 \\ 0 & if\ \Delta\rho(r) < 0 \end{matrix} \right\} \quad \text{....................... (S3)}$$

$$\rho_-(r) = \left\{ \begin{matrix} \Delta\rho(r) & if\ \Delta\rho(r) < 0 \\ 0 & if\ \Delta\rho(r) > 0 \end{matrix} \right\} \quad \text{........................ (S4)}$$

The barycenters of the spatial regions R_+ and $R_–$ are related with $\rho_+(r)$ and $\rho_-(r)$ and are shown as

$$R_+ = \frac{\int r\rho_+(r)dr}{\int \rho_+(r)dr} = (x_+, y_+, z_+) \quad \text{.................... (S5)}$$

$$R_- = \frac{\int r\rho_-(r)dr}{\int \rho_-(r)dr} = (x_-, y_-, z_-) \quad \text{...................... (S6)}$$

The spatial distance (D_{CT}) between the two barycenters R_+ and R_- of density distributions can thus be used to measure the CT excitation length

$$D_{CT} = |R_+ - R_-| \qquad \text{(S7)}$$

The transferred charge (q_{CT}) can be obtained by integrating over all space $\rho_+ (\rho_-)$,. Variation in dipole moment between the ground and the excited states (μ_{CT}) can be computed by the following relation:

$$\|\mu_{CT}\| = D_{CT}\int \rho_+(r)dr = D_{CT}\int \rho_-(r)dr \qquad \text{(S8)}$$

$$= D_{CT}q_{CT} \qquad \text{(S9)}$$

The difference between the dipole moments $\|\mu_{CT}\|$ have been computed for the ground and the excited states $\Delta\mu_{ES-GS}$. The two centroids of charges (C^+/C^-) associated to the positive and negative density regions are calculated as follows. First the root–mean–square deviations along the three axis (σ_{aj}, j = x, y, z; a = + or –) are computed as

$$\sigma_{a,j} = \sqrt{\frac{\sum_i \rho_a(r_i)(j_i - j_a)^2}{\sum_i \rho_a(r_i)}} \qquad \text{(S10)}$$

The two centroids (C_+ and C_-) are defined as

$$C_+(r) = A_+ e\left(-\frac{(x - x_+)^2}{2\sigma_{+x}^2} - \frac{(y - y_+)^2}{2\sigma_{+y}^2} - \frac{(z - z_+)^2}{2\sigma_{+z}^2}\right) \qquad \text{(S11)}$$

$$C_{-+}(r) = A_- e\left(-\frac{(x - x_-)^2}{2\sigma_{-x}^2} - \frac{(y - y_-)^2}{2\sigma_{-y}^2} - \frac{(z - z_-)^2}{2\sigma_{-z}^2}\right) \qquad \text{(S12)}$$

The normalization factors (A+ and A_) are used to impose the integrated charge on the centroid to be equal to the corresponding density change integrated in the whole space:

$$A_{+} = \frac{\int \rho_{+}(r)dr}{\int e\left(-\frac{(x-x_{-})^2}{2\sigma_{+x}^2}-\frac{(y-y_{-})^2}{2\sigma_{+y}^2}-\frac{(z-z_{-})^2}{2\sigma_{+z}^2}\right)dr} \quad \text{.................. (S13)}$$

$$A_{-} = \frac{\int \rho_{-}(r)dr}{\int e\left(-\frac{(x-x_{-})^2}{2\sigma_{-x}^2}-\frac{(y-y_{-})^2}{2\sigma_{-y}^2}-\frac{(z-z_{-})^2}{2\sigma_{-z}^2}\right)dr} \quad \text{.................. (S14)}$$

H index is defined as half of the sum of the centroids axis along the D–A direction, if the D–A direction is along the X axis, H is defined by the relation:

$$H = \frac{\sigma_{+x} + \sigma_{-x}}{2} \quad \text{.................... (S15)}$$

The centroid along X axis is expected. The t intex represents the difference between D_{CT} and H:

$$t = D_{CT} - H \quad \text{............... (S16)}$$

SI- XVII: Figures

Figure S1. ^{1}H NMR spectrum of 4'-(1-(naphthalen-1-yl)-1H-phenanthro[9,10-d]imidazol-2-yl)-N,N-diphenyl-(2-[1,1'-biphenyl]vinyl)-4-amine (NSPI-TPA)

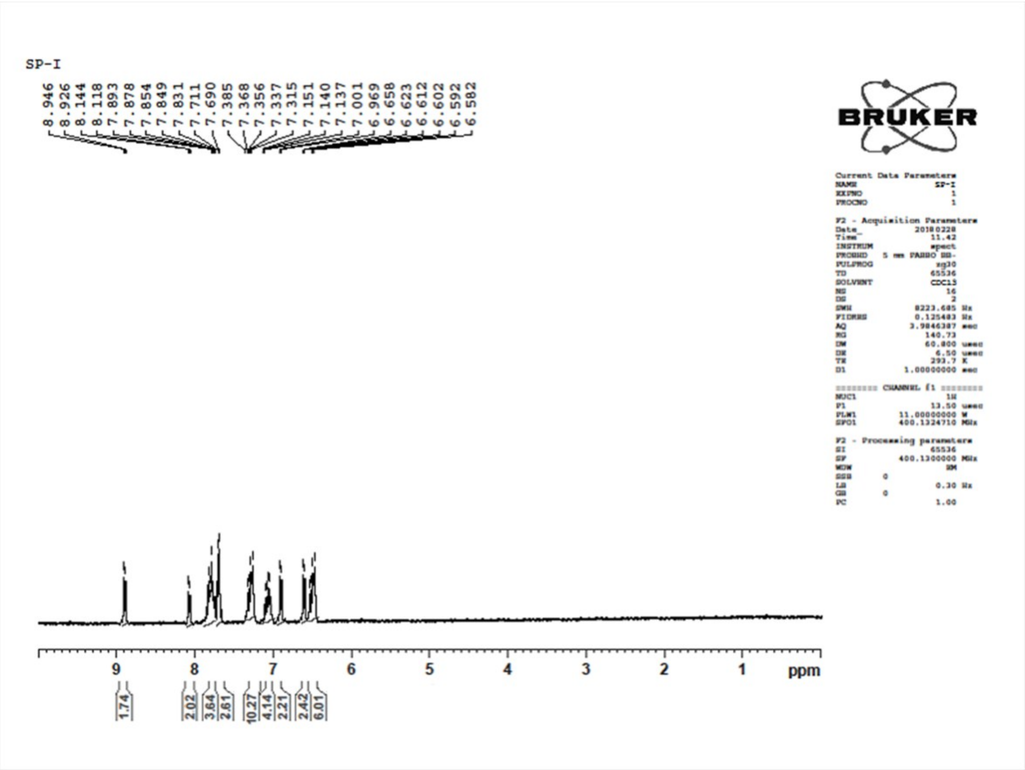

Figure S2. ^{13}C NMR spectrum of 4'-(1-(naphthalen-1-yl)-1H-phenanthro[9,10-d]imidazol-2-yl)-N,N-diphenyl-(2-[1,1'-biphenyl]vinyl)-4-amine (NSPI-TPA)

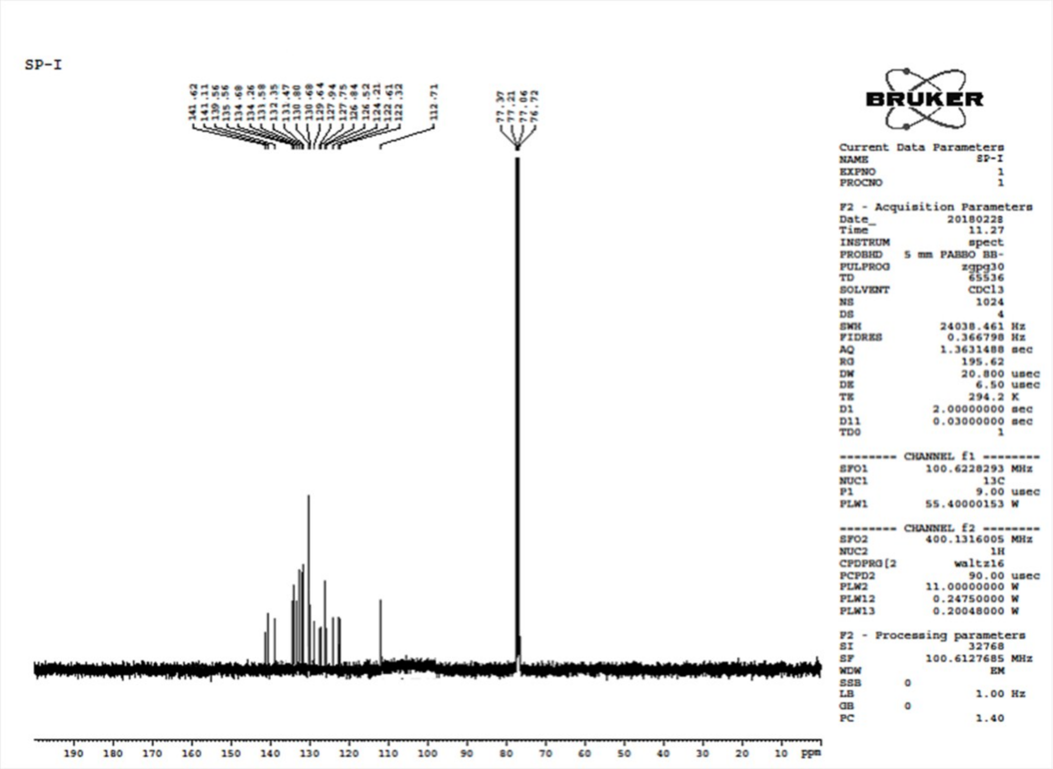

Figure S3. ^{1}H NMR spectrum of 4'-(1-(2-methylnaphthalen-1-yl)-1H- phenanthro[9,10-d]imidazol-2-yl)-N,N-diphenyl-(2-[1,1'-biphenyl]vinyl-4-amine (MNSPI-TPA)

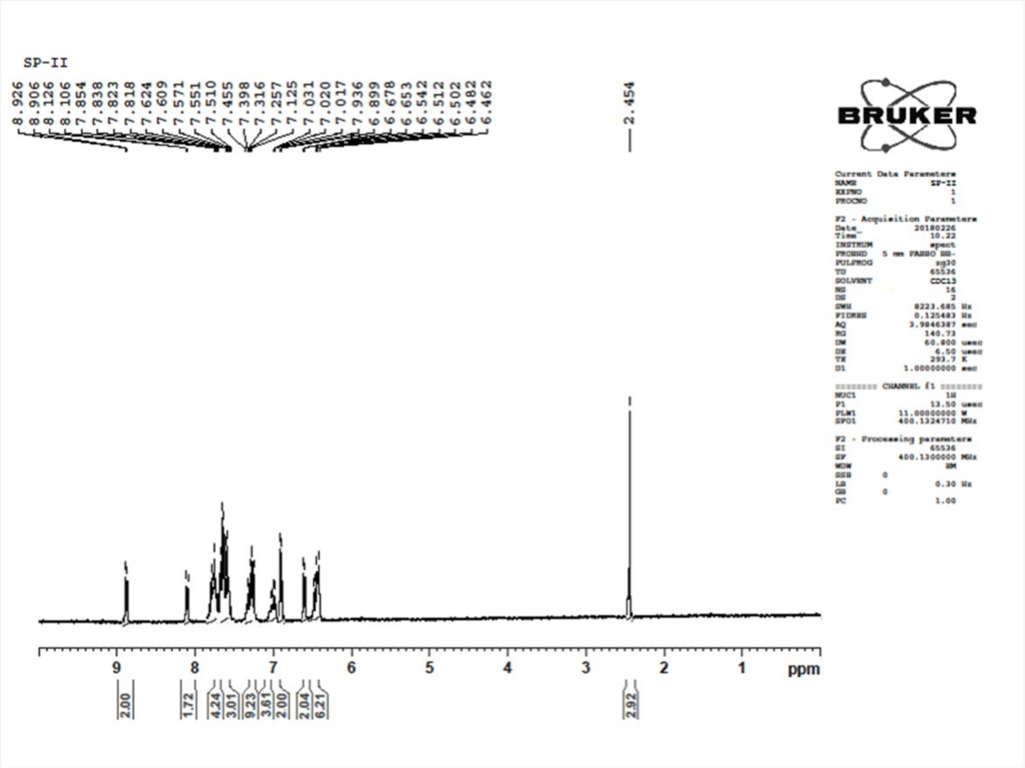

Figure S4. ^{13}C NMR spectrum of 4'-(1-(2-methylnaphthalen-1-yl)-1H- phenanthro[9,10-d]imidazol-2-yl)-N,N-diphenyl-(2-[1,1'-biphenyl]vinyl-4-amine (MNSPI-TPA)

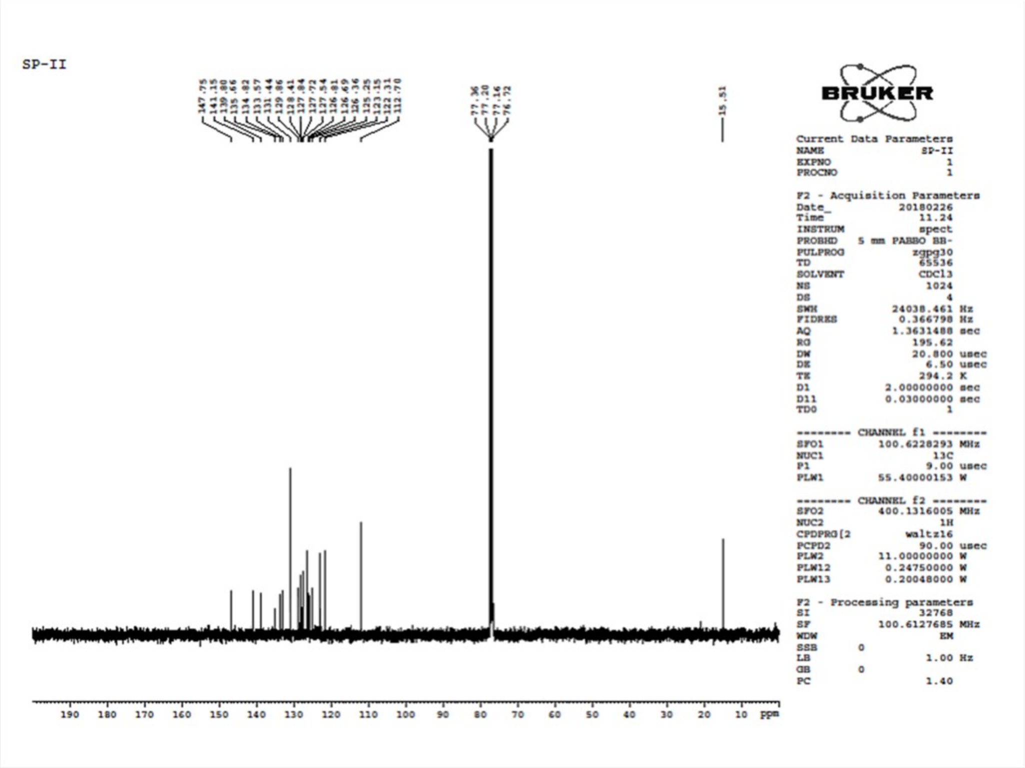

Figure S5. ^{1}H NMR spectrum of 4-(2-(4'-(diphenylamino)-(2-[1,1'-biphenyl]vinyl-4-yl)-1H-phenanthro[9,10-d]imidazol-1-yl)-1-naphthonitrile (SPNCN-TPA)

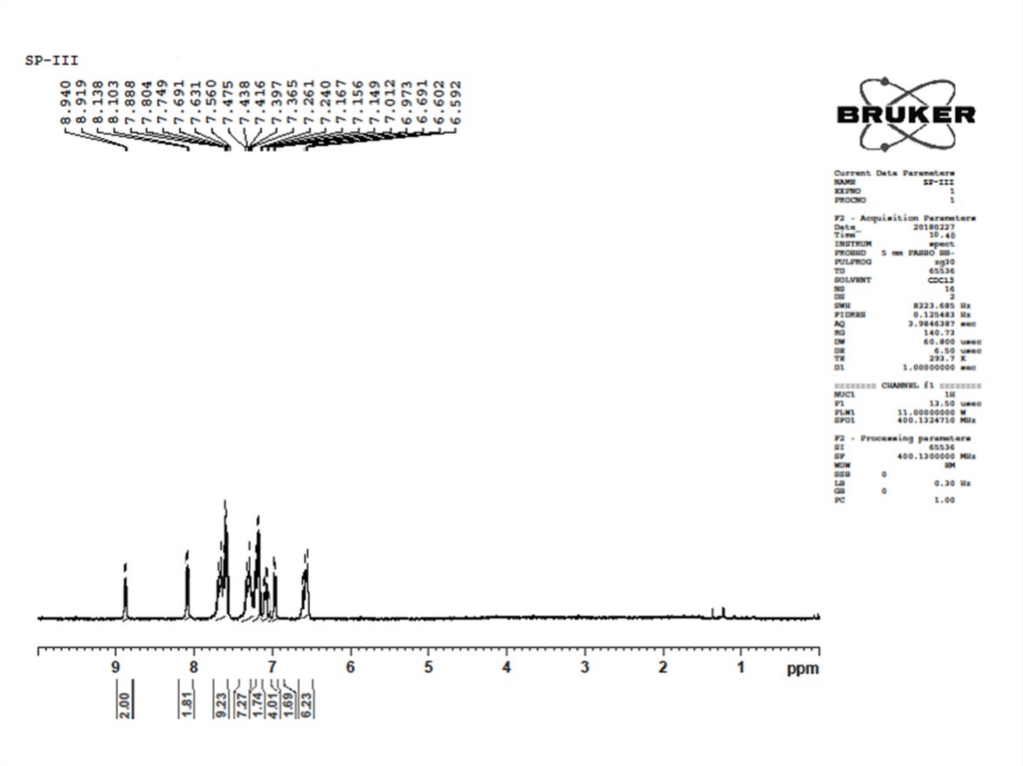

Figure S6. ^{13}C NMR spectrum of 4-(2-(4'-(diphenylamino)-(2-[1,1'-biphenyl]vinyl-4-yl)-1H-phenanthro[9,10-d]imidazol-1-yl)-1-naphthonitrile (SPNCN-TPA)

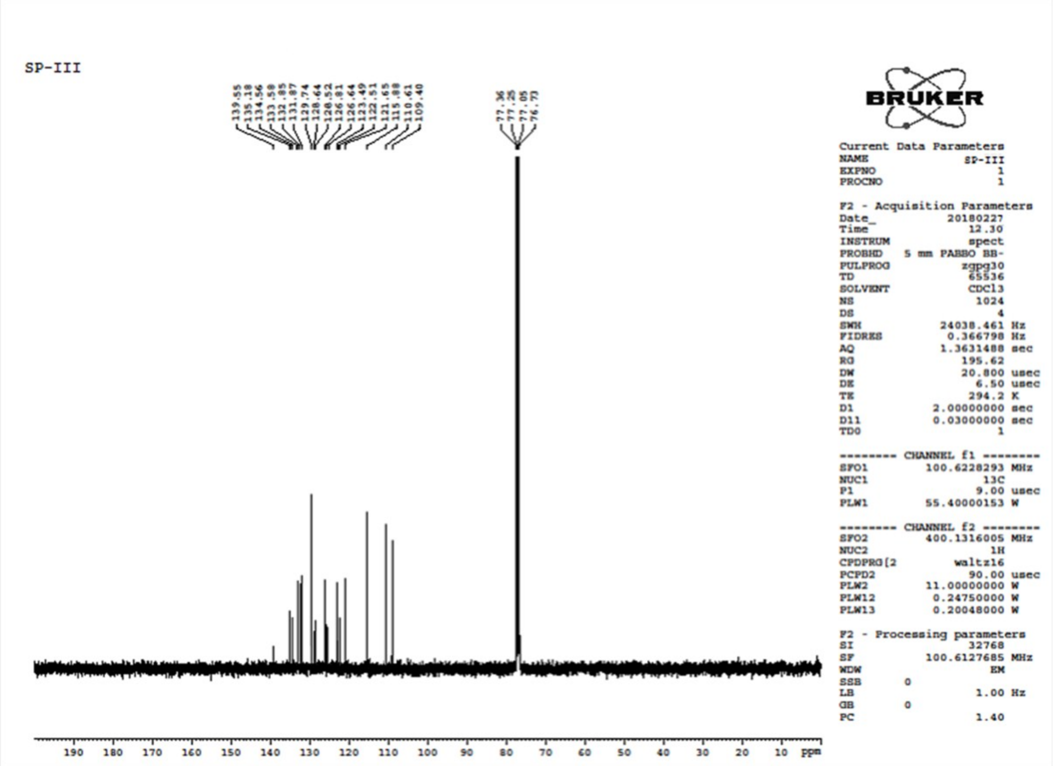

Figure S7. ^{1}H NMR spectrum of 4-(2-(4-(9H-carbazol-9-yl)styryl)-1H-phenanthro[9,10-d]imidazol-1-yl)naphthalene-1-carbonitrile (SPNCN-Cz)

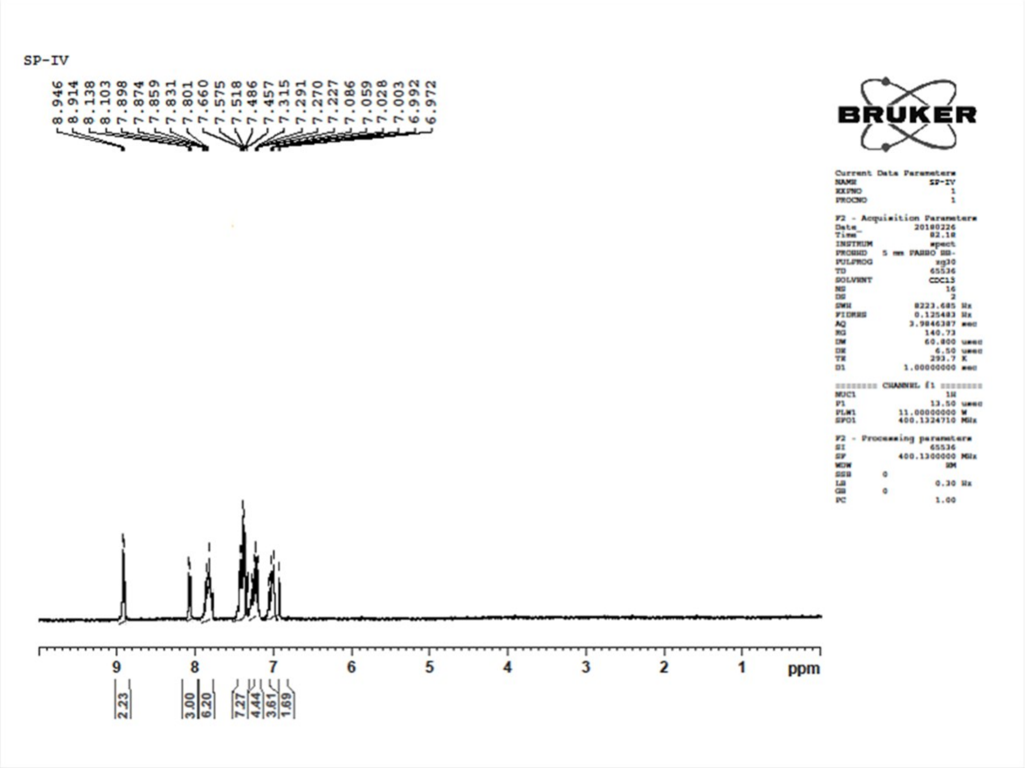

Figure S8. ^{13}C NMR spectrum of 4-(2-(4-(9H-carbazol-9-yl)styryl)-1H-phenanthro[9,10-d]imidazol-1-yl)naphthalene-1-carbonitrile (SPNCN-Cz)

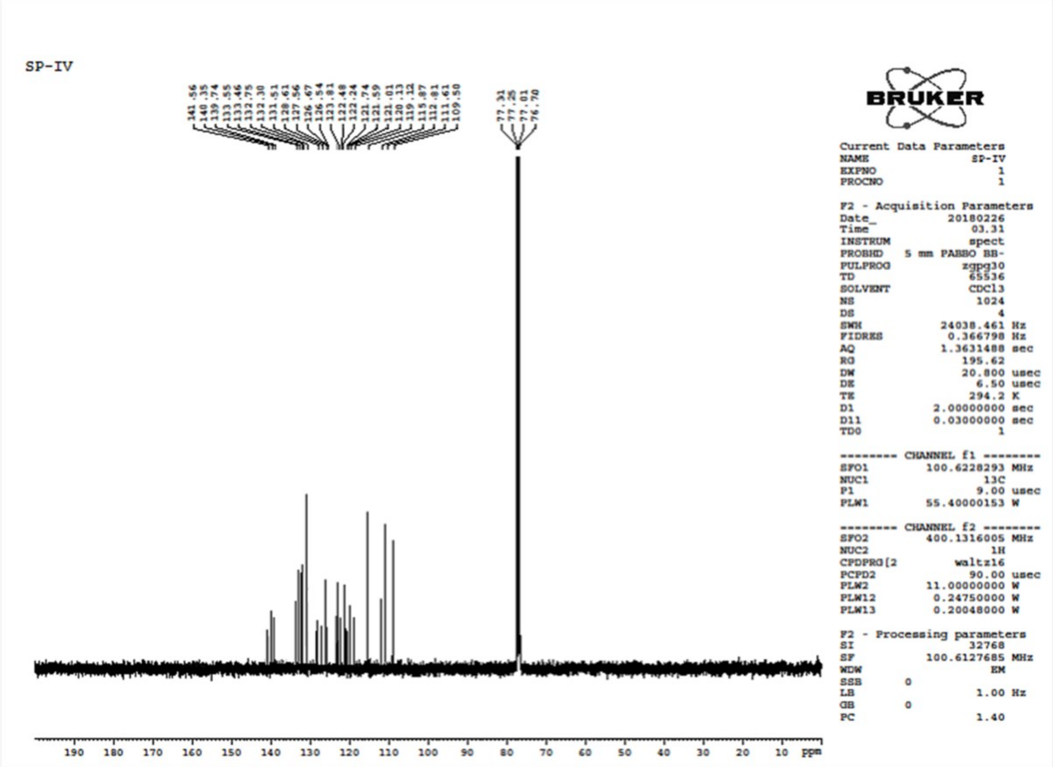

Figure S9. MALDI-TOF mass spectra of (a) NSPI-TPA; (b) MNSPI-TPA; (c) SPNCN-TPA and (d) SPNCN-Cz

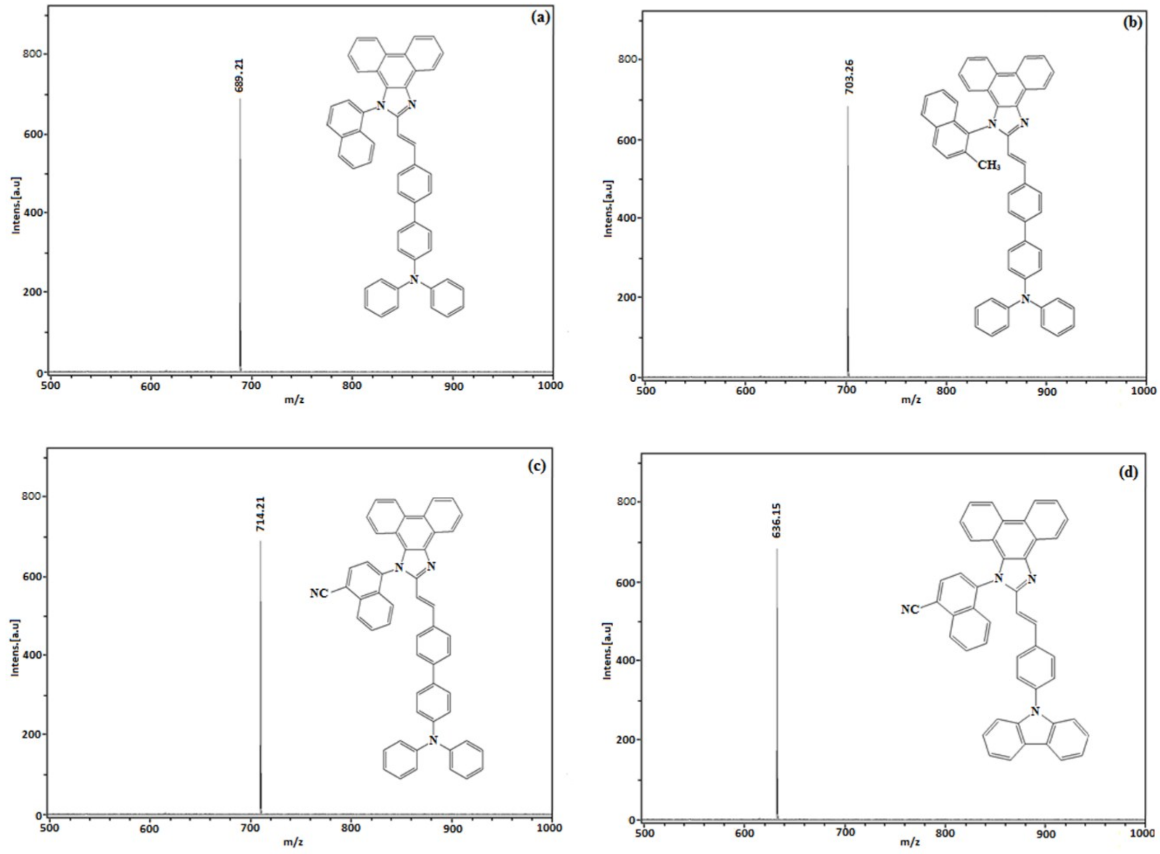

Figure S10. Solvatochromic emission spectra of a) SPICN-TPA and b) SPICN-Cz

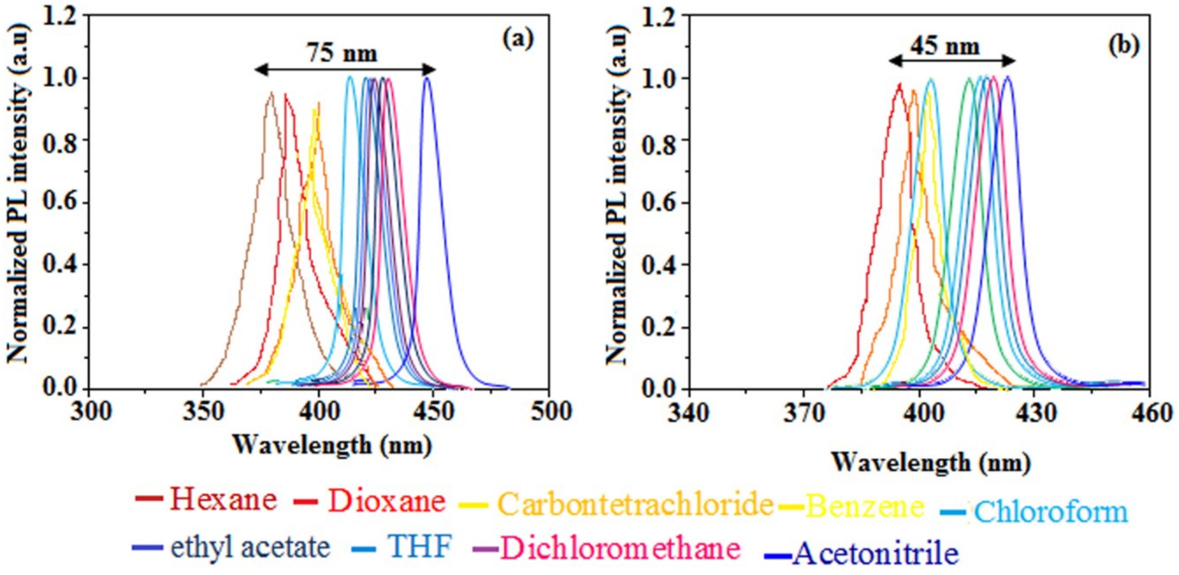

Figure S11. Normalized absorption spectra of a) SPICN-TPA and b) SPICN-Cz

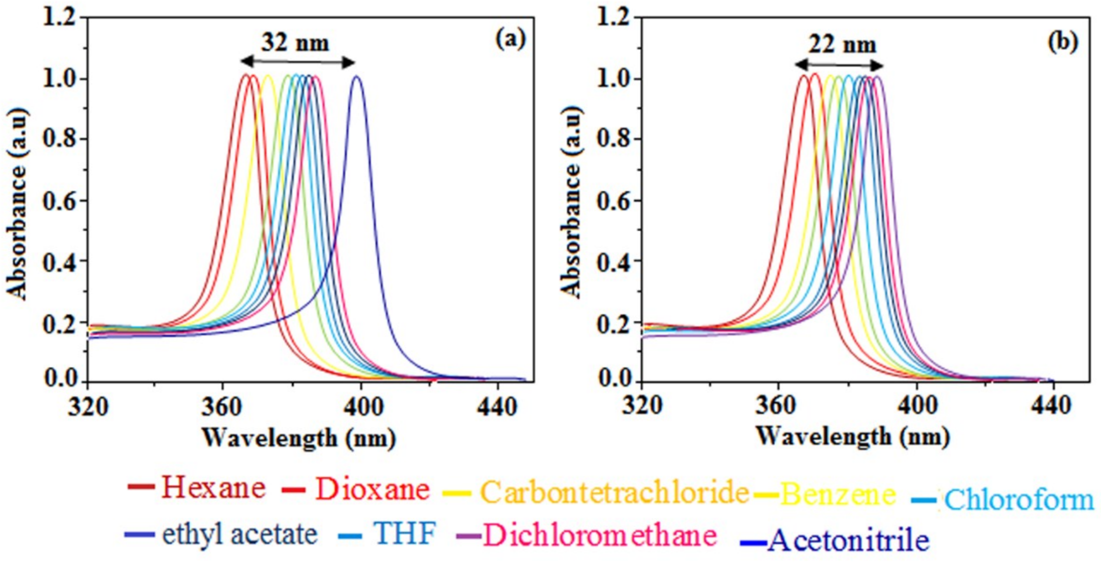

Figure S12. Computed hole and particle distribution of SPNCN-TPA for first ten single states (S_1–S_{10}); green and blue areas represent increasing and decreasing electron density respectively.

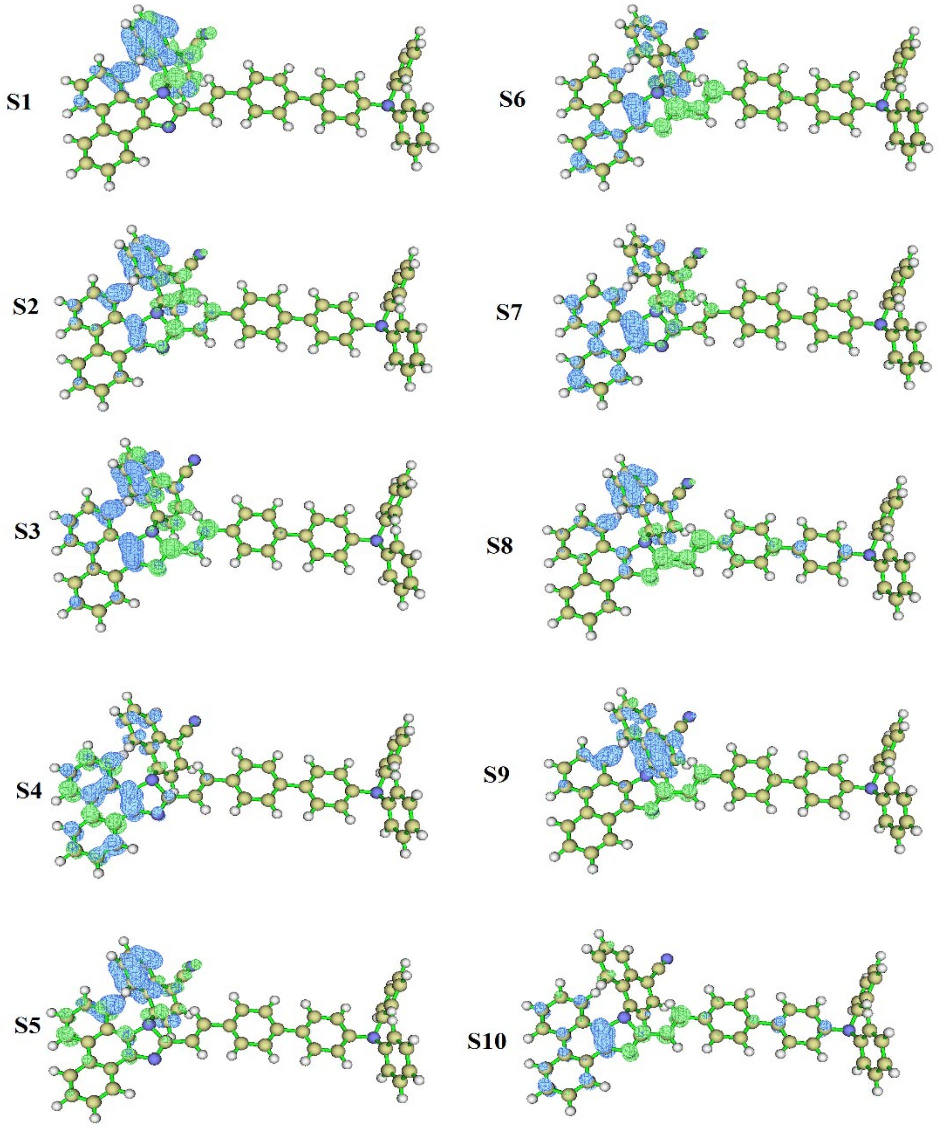

Figure S13. Computed hole and particle distribution of SPNCN-Cz for first ten single states (S_1–S_{10}); green and blue areas represent increasing and decreasing electron density respectively.

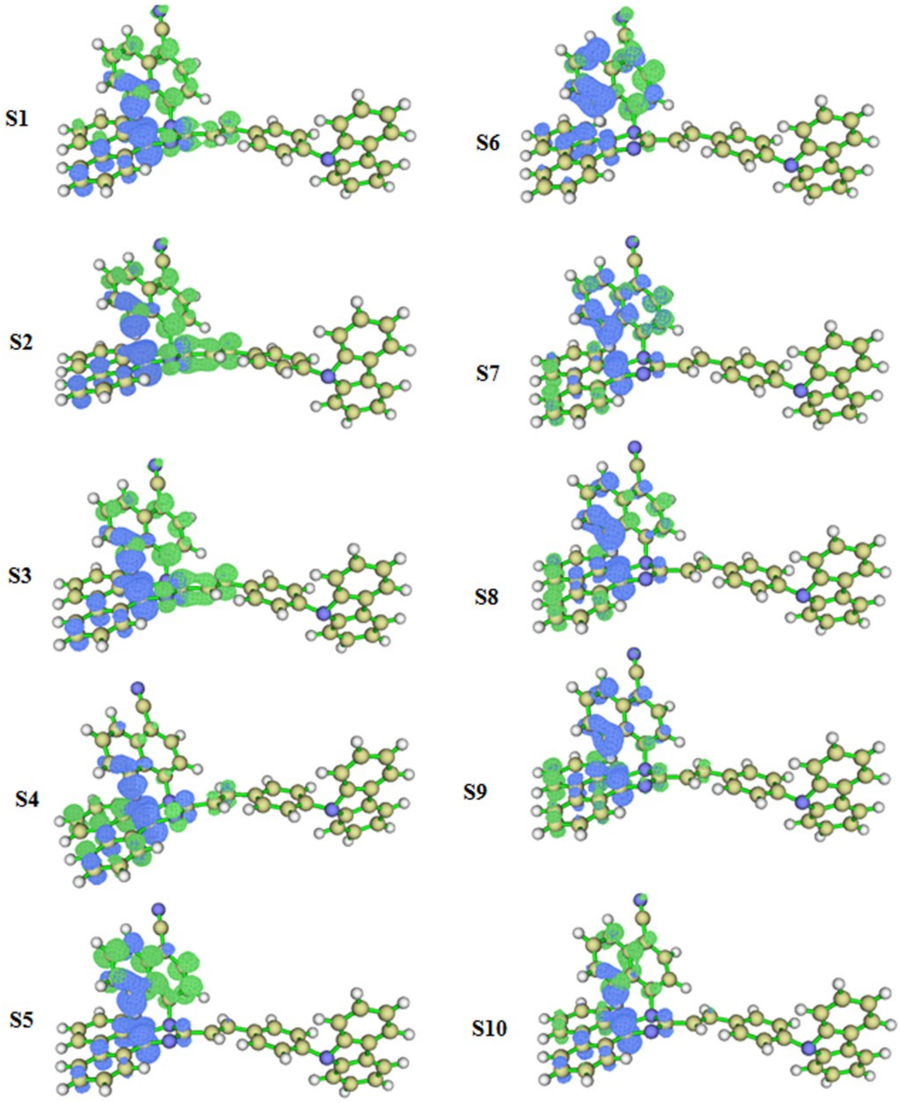

Figure S14. Computed natural transition orbital (NTO) pairs and transition character analysis for the first ten singlet (S_1-S_{10}) and triplet states (T_1-T_{10}) of SPNCN-TPA [*f*-oscillator strength and percentage weights of hole-particle are given for $S_0 \rightarrow S_n$ and $S_0 \rightarrow T_n$ transitions (n=1-10)]

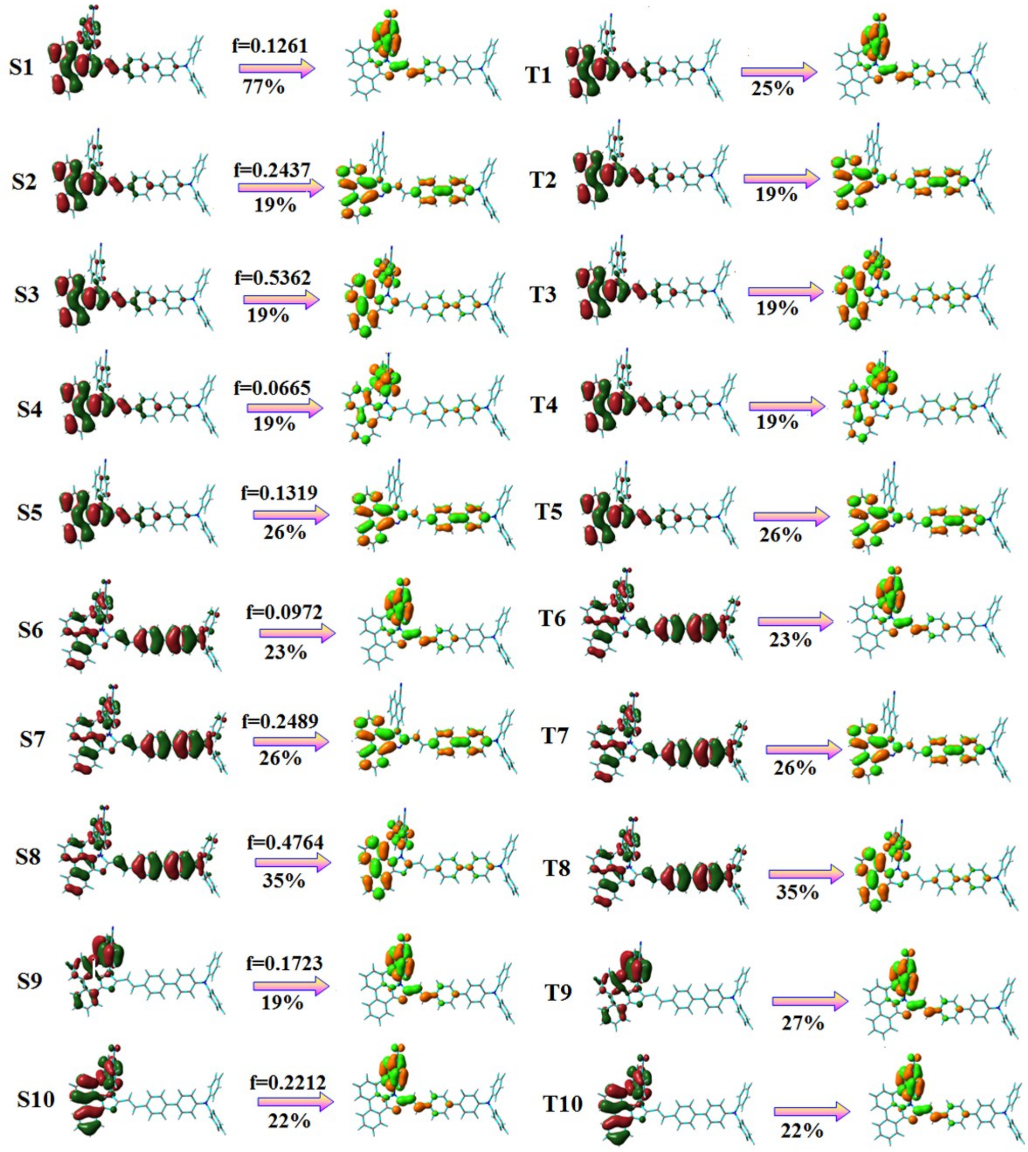

Figure S15. Computed natural transition orbital (NTO) pairs and transition character analysis for the first ten singlet (S_1-S_{10}) and triplet states (T_1-T_{10}) of SPNCN-Cz [*f*-oscillator strength and percentage weights of hole-particle are given for $S_0 \rightarrow S_n$ and $S_0 \rightarrow T_n$ transitions (n=1-10)]

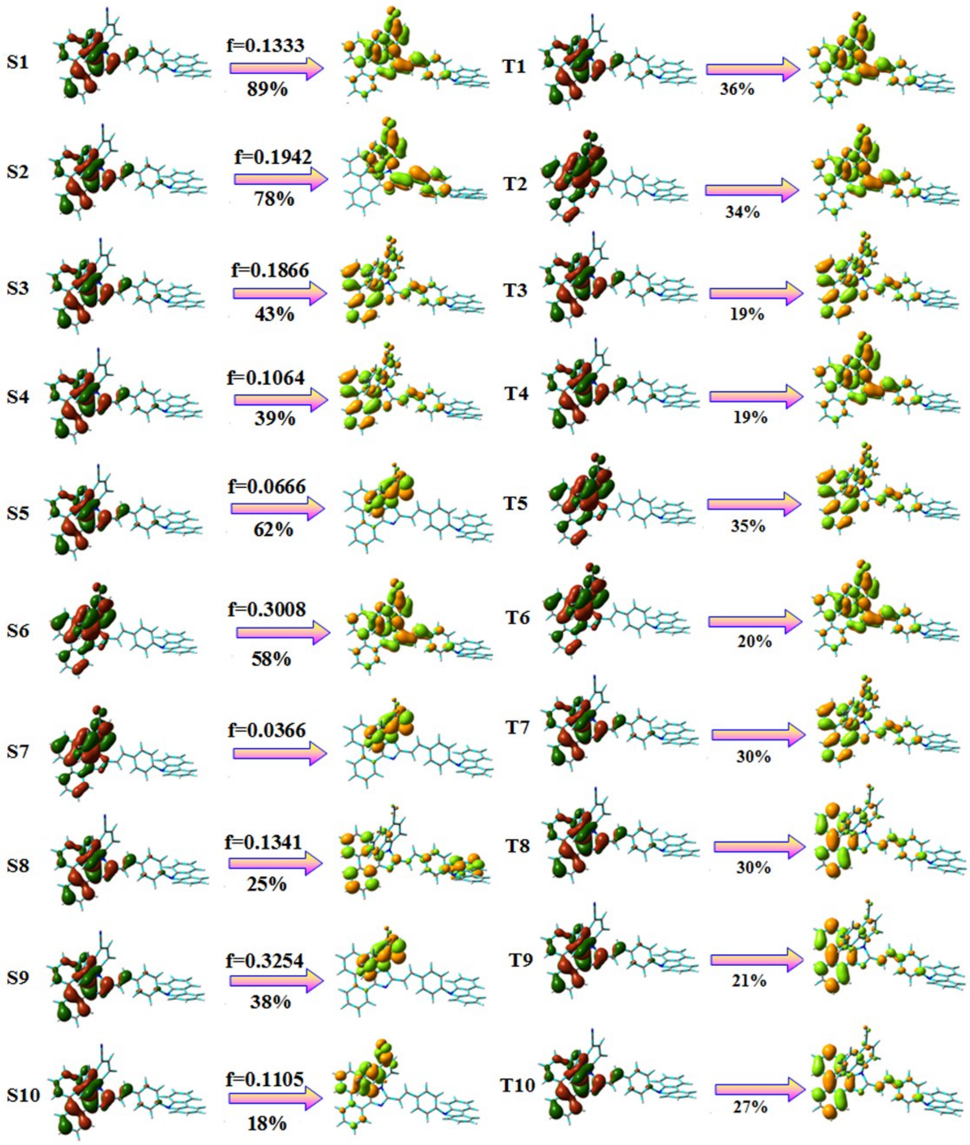

Figure S16: Computed contour plots of transition density matrices (TDM) of SPNCN-TPA for S_1–S_5 states.

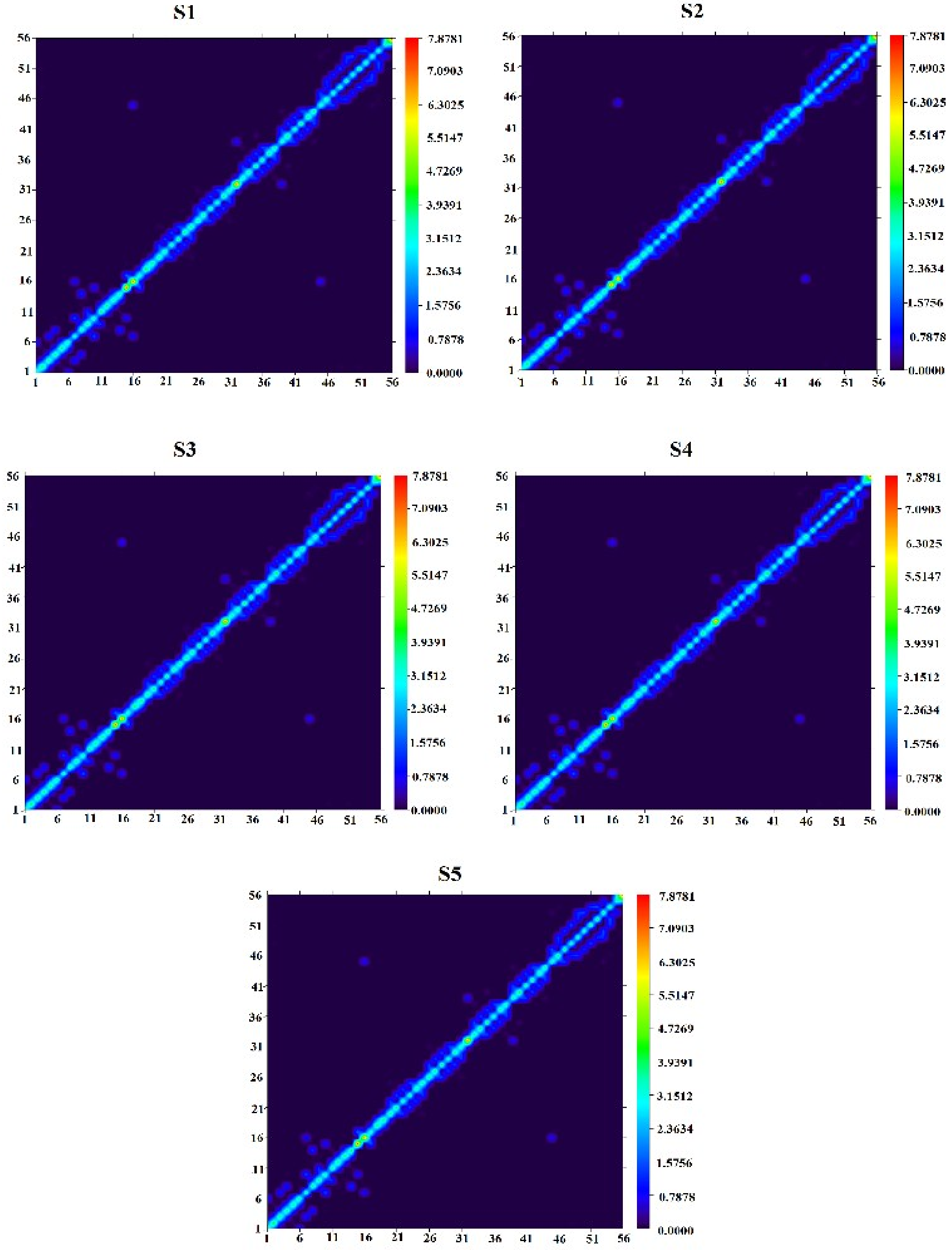

Figure S17: Computed contour plots of transition density matrices (TDM) of SPNCN-Cz for S_1–S_5 states.

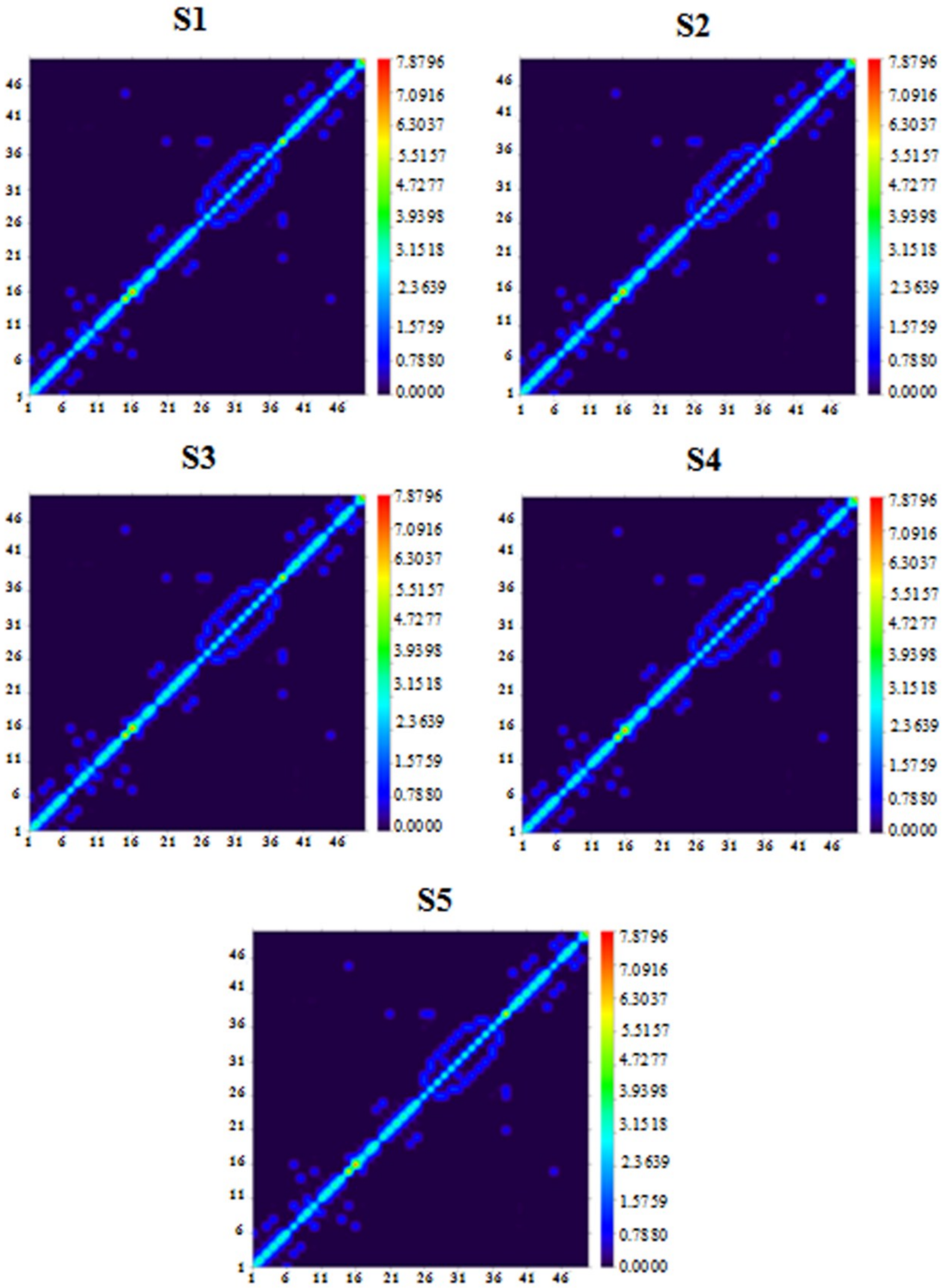

SI-X: Tables

Table S1: Detailed photophysical properties of SPNCN-TPA in different solvents.

Solvents	ε	n	f(ε,n)	ET(30)	λ_{ab} (nm)	ν_{ab} (cm^{-1})	λ_{flu} (nm)	ν_{flu} (cm^{-1})	ν_{ss} (cm^{-1})	ΔG (kcal/mol)	$\Delta(\Delta G_{hex}-\Delta G_{sol})$ (kcal/mol)	λ (kcal/mol)
Hexane	1.88	1.37	0.000411	32.4	366	27322.4	373	26455.03	1100.4	76.86	5.66	1.24
Dioxane	2.22	1.42	0.021437	36.0	369	27100.2	385	25974.03	1126.2	75.86	6.66	1.61
Carbontetrachloride	2.23	1.46	0.011075	39.1	372	26881.7	390	25641.03	1200.0	75.07	7.45	1.77
Benzene	2.28	1.42	0.026639	34.3	378	26455.0	391	25575.45	1300.9	74.36	8.16	1.26
Chloroform	4.81	1.44	0.148262	39.1	387	25839.7	410	24390.24	1941.6	71.79	10.73	2.07
Ethyl acetate	6.09	1.41	0.186569	38.1	383	26109.6	415	24096.39	1449.5	71.76	10.76	2.88
THF	7.52	1.40	0.209634	37.4	383	26109.6	431	23201.86	1500.1	70.48	12.04	4.16
Dichloromethane	9.08	1.42	0.218349	40.7	398	25125.6	448	22321.43	1800.2	67.81	14.71	4.01
Acetonitrile	37.5	1.34	0.305378	45.6	367	27247.9	422	23696.68	1300.8	72.81	9.71	5.08

Table S2: Detailed photophysical properties of SPNCN-Cz in different solvents.

Solvents	ε	n	f(ε,n)	ET(30)	λ_{ab} (nm)	ν_{ab} (cm^{-1})	λ_{flu} (nm)	ν_{flu} (cm^{-1})	ν_{ss} (cm^{-1})	ΔG (kcal/mol)	$\Delta(\Delta G_{hex}-\Delta G_{sol})$ (kcal/mol)	λ (kcal/mol)
Hexane	1.88	1.37	0.000411	32.4	368	27173.91	390	25641.03	1200.7	75.49	7.03	2.19
Dioxane	2.22	1.42	0.021437	36.0	367	27247.96	394	25380.71	1310.9	75.22	7.30	2.67
Carbontetrachloride	2.23	1.46	0.011075	39.1	375	26666.67	400	25000.00	1200.8	73.84	8.68	2.38
Benzene	2.28	1.42	0.026639	34.3	379	26385.22	402	24875.62	1509.6	73.26	9.26	2.16
Chloroform	4.81	1.44	0.148262	39.1	386	25906.74	420	23809.52	1610.2	71.06	11.46	3.00
Ethyl acetate	6.09	1.41	0.186569	38.1	380	26315.79	425	23529.41	1600.1	71.24	11.28	3.98
THF	7.52	1.40	0.209634	37.4	389	25706.94	428	23364.49	2000.5	70.14	12.38	3.35
Dichloromethane	9.08	1.42	0.218349	40.7	390	25641.03	436	22935.78	2342.4	69.43	13.09	3.87
Acetonitrile	37.5	1.34	0.305378	45.6	375	26666.67	427	23419.20	1210.7	71.59	10.93	4.64

Table S3: Computed excitation energy (eV), excitation coefficient and Δr intex (Å) for first ten singlet & triplet states of SPNCN-TPA.

State	Singlet			Triplet		
	Excitation energy	Excitation coefficient	Δr intex	Excitation energy	Excitation coefficient	Δr intex
1	1.8564	0.4512	2.6902	1.0337	0.3419	2.8747
2	2.5982	0.3841	2.9476	1.4621	0.2704	3.3012
3	3.2337	0.4265	3.0228	1.8564	0.2411	3.1288
4	2.2089	0.3886	2.5989	1.8984	0.2698	4.4636
5	3.5790	0.3748	2.8390	1.9372	0.2582	3.5922
6	3.6604	0.4011	4.3462	1.9800	0.3376	2.5547
7	3.8563	0.3880	2.7934	2.0141	0.3426	2.4375
8	3.8697	0.3929	3.6828	2.2448	0.2097	3.2220
9	2.3083	0.3718	5.1501	2.4031	0.3382	2.9111
10	4.0491	0.3330	4.2253	2.4208	0.2508	2.7478

Table S4: Computed excitation energy (eV), excitation coefficient and Δr intex (Å) for first ten singlet & triplet states of SPNCN-Cz.

State	Singlet			Triplet		
	**Excitation energy**	**Excitation coefficient**	**Δr intex**	**Excitation energy**	**Excitation coefficient**	**Δr intex**
1	1.5056	0.4581	2.1166	0.8911	0.4083	1.8451
2	1.4843	0.4334	1.8085	1.2676	0.3279	3.3501
3	2.7449	0.4279	1.9804	1.5056	0.2747	3.5916
4	1.6373	0.4368	4.3566	1.8630	0.2403	2.9946
5	3.3437	0.4330	3.1627	2.0033	0.3884	2.6931
6	3.4154	0.4198	3.1581	2.0033	0.2312	3.2011
7	3.6786	0.3973	2.0510	2.1570	0.2903	2.7894
8	3.7524	0.3489	2.6077	2.2857	0.3717	3.2941
9	3.8573	0.3549	3.0051	2.3497	0.2736	2.5449
10	3.9054	0.3630	3.9147	2.4017	0.1471	3.5162

Table S5: Computed hole (H) and electron (E) overlap (S), distance between centroids of H and E (D, Å) and dipole moment (μ) for the first ten S_1–S_{10} states of SPNCN-TPA.

State	Integral of hole	Integral of electron	Integral of transition density	Integral overlap of H–E (S)	Centroid of H (Å)			Centroid of E (Å)			D (Å)	μ (a.u)
					x	y	z	x	y	z		
S1	0.9006	0.7263	-0.0021	0.3835	-5.6293	2.1749	0.6300	-4.5203	2.2475	0.0541	1.25	1.9243
S2	0.7765	0.5902	0.0204	0.3309	-5.1512	0.8468	0.3953	-4.2221	0.9279	-0.0972	1.05	1.3621
S3	0.8647	0.6622	0.0008	0.3575	-5.2417	0.5025	0.3598	-3.9985	0.6323	0.1451	1.27	1.8299
S4	0.8009	0.6010	0.0027	0.3952	-6.0712	-0.6332	0.1841	-6.4606	-0.5931	-0.0207	0.44	0.5852
S5	0.7565	0.5757	0.0116	0.3308	-5.4025	1.6253	0.5164	-5.5919	0.8637	0.0076	0.94	1.1772
S6	0.8378	0.6492	0.0102	0.3867	-4.5351	0.0259	0.0125	-3.6480	0.5372	0.1201	1.03	1.4466
S7	0.8028	0.6025	0.0137	0.4142	-5.7240	-0.6599	0.1073	-4.8177	-0.0280	-0.0745	1.12	1.4868
S8	0.8048	0.6256	-0.0001	0.3311	-3.1777	0.9145	0.2532	-2.3984	0.0722	0.0207	1.17	1.5825
S9	0.7751	0.5943	-0.0081	0.3957	-4.8453	1.0955	0.1047	-4.1389	0.6574	0.1115	0.83	1.0757
S10	0.7005	0.5222	-0.0113	0.3377	-3.3424	-0.7305	-0.0340	-3.2549	-0.4343	0.1016	0.3	0.3897

Table S6: Computed RMSD of electron and hole, H index and t index for first ten singlet states (S_1–S_{10}) of SPNCN-TPA.

State	RMSD of electron				RMSD of hole				H index				t index			
	**x**	**y**	**z**	**total**	**x**	**y**	**z**	**total**	**x**	**y**	**z**	**norm**	**x**	**y**	**z**	**norm**
S1	1.761	1.921	1.263	2.896	1.787	1.929	1.137	2.865	1.774	1.925	1.200	2.880	-0.665	-1.853	-0.624	2.065
S2	2.297	2.385	1.167	3.511	3.292	2.478	1.029	4.247	2.795	2.432	1.098	3.864	-1.866	-2.351	-0.605	3.061
S3	2.505	2.319	1.067	3.576	2.977	2.547	1.023	4.050	2.741	2.433	1.045	3.811	-1.498	-2.303	-0.830	2.870
S4	2.882	2.639	0.885	4.007	2.835	2.607	0.913	3.958	2.858	2.623	0.899	3.982	-2.469	-2.583	-0.694	3.640
S5	2.745	2.551	1.070	3.897	2.538	2.275	1.093	3.579	2.641	2.413	1.082	3.738	-2.452	-1.652	-0.573	3.011
S6	2.719	2.303	1.022	3.707	3.848	2.640	0.985	4.770	3.284	2.472	1.003	4.230	-2.396	-1.960	-0.896	3.223
S7	3.157	2.632	0.971	4.223	3.233	2.562	0.914	4.225	3.195	2.597	0.943	4.224	-2.289	-1.965	-0.761	3.111
S8	3.823	2.200	0.935	4.509	5.018	2.381	1.097	5.662	4.421	2.291	1.016	5.081	-3.641	-1.448	-0.783	3.996
S9	2.974	2.419	1.068	3.980	2.907	2.230	1.015	3.802	2.940	2.325	1.041	3.890	-2.234	-1.886	-1.035	3.102
S10	4.325	2.316	0.882	4.984	5.567	2.262	0.869	6.072	4.946	2.289	0.875	5.520	-4.859	-1.992	-0.740	5.303

Table S7: Computed hole (H) and electron (E) overlap (S), distance between centroids of H and E (D, Å) and dipole moment (μ) for the first ten S_1–S_{10} states of SPNCN-Cz.

State	Integral of hole	Integral of electron	Integral of transition density	Integral overlap of H–E (S)	Centroid of H (Å)			Centroid of E (Å)			D (Å)	μ (a.u)
					x	y	z	x	y	z		
S1	0.8359	0.7214	-0.0150	0.2331	-3.77	-1.06	0.15	-2.84	0.93	0.01	2.20	3.24
S2	0.8505	0.7185	0.0123	0.2453	-3.77	-1.06	0.15	-2.41	0.90	0.07	2.39	3.54
S3	0.8505	0.7185	0.0123	0.2453	-3.77	-1.06	0.15	-2.41	0.90	0.07	2.39	3.54
S4	0.8056	0.6473	0.0133	0.2538	-3.77	-1.06	0.15	-4.20	-1.03	-0.22	0.56	0.77
S5	0.8581	0.6533	0.0008	0.2342	-4.11	-0.21	0.26	-3.83	2.10	0.46	2.34	3.34
S6	0.8748	0.6685	-0.0038	0.4122	-4.43	0.77	0.28	-3.59	2.18	0.17	1.64	2.39
S7	0.8264	0.6028	-0.0131	0.4341	-3.83	0.43	0.21	-4.17	0.71	0.01	0.48	0.65
S8	0.8022	0.5971	0.0107	0.3879	-3.93	-0.03	0.21	-4.12	-0.19	-0.22	0.50	0.67
S9	0.7887	0.5760	-0.0178	0.3776	-3.73	-0.02	0.12	-4.08	-0.41	-0.45	0.77	1.00
S10	0.6858	0.4973	0.0062	0.2626	-3.72	-0.78	0.12	-4.34	0.89	0.17	1.79	2.00

Table S8: Computed RMSD of electron and hole, H index and t index for first ten singlet states (S_1–S_{10}) of SPNCN-Cz.

State	RMSD of electron				RMSD of hole				H index				t index			
	x	y	z	total	x	y	z	total	x	y	z	norm	x	y	z	Norm
S1	2.629	2.290	1.156	3.673	1.779	1.736	1.038	2.693	2.204	2.013	1.097	3.180	-1.275	-0.021	-0.959	1.596
S2	2.352	2.108	1.038	3.325	1.779	1.736	1.038	2.693	2.065	1.922	1.038	3.006	-0.706	0.047	-0.959	1.192
S3	2.352	2.108	1.038	3.325	1.779	1.736	1.038	2.693	2.065	1.922	1.038	3.006	-0.706	0.047	-0.959	1.192
S4	2.490	2.388	1.258	3.672	1.779	1.736	1.038	2.693	2.134	2.062	1.148	3.182	-1.712	-2.035	-0.776	2.770
S5	1.660	1.550	1.283	2.608	1.637	2.194	1.185	2.983	1.649	2.231	1.022	5.310	-1.373	0.447	-1.027	1.771
S6	1.667	2.179	1.174	2.985	1.642	2.389	1.341	3.194	1.654	2.284	1.258	3.088	-0.818	-0.875	-1.142	1.655
S7	2.230	2.829	1.323	3.837	2.244	2.333	1.247	3.469	2.237	2.581	1.285	3.649	-1.893	-2.306	-1.088	3.175
S8	2.662	2.669	1.288	3.983	2.321	2.329	1.258	3.521	2.491	2.499	1.273	3.751	-2.307	-2.338	-0.828	3.387
S9	2.633	2.328	2.328	3.715	2.688	2.312	1.255	3.761	2.661	2.320	1.229	3.738	-2.307	-1.936	-0.654	3.082
S10	2.025	2.500	1.311	3.473	2.461	2.053	1.166	3.410	2.243	2.276	1.238	3.427	-1.623	-0.596	-1.186	2.096

Table S9: Barycentres of electron density loss (R_+) /gain (R-), distance between two barycenters (D_{CT}), RMSD of +ve/-ve parts, CT indices (H & t) and overlap integral of C+/C- of SPNCN-TPA and SPNCN-Cz.

Blue Fluorescent emissive materials	R_+ (Å)			R- (Å)			D_{CT} (Å)	RMSD of +ve parts	RMSD of -ve parts	H / t indices (Å)	overlap integral of C+/ C-
	X	y	Z	x	y	z					
SPNCN-TPA	-0.23	-0.28	-0.001	-1.128	-0.354	-0.028	1.123	13.23	13.41	6.262/ 5.633	0.9795
SPNCN-Cz	-0.36	-0.56	-0.180	-1.311	-0.273	-0.164	0.964	10.53	11.23	5.664/ 4.725	0.9873

Table S10. Comparison of device efficiencies with reported non-doped emitters.

Emitter	V^{α}_{on}(V)	L(cd/m^2)	EL(nm)	η_c(cd/A)	η_p(lm/W)	CIE(x,y)	ref
**NSPI-TPA**	**4.5**	**2013**	**438**	**1.38**	**1.29**	**(0.15, 0.09)**	**This work**
**MNSPI-TPA**	**4.3**	**2158**	**447**	**1.68**	**1.51**	**(0.15, 0.08)**	**This work**
**SPNCN-TPA**	**3.8**	**4362**	**451**	**2.32**	**2.01**	**(0.15, 0.05)**	**This work**
**SPNCN-Cz**	**3.5**	**4826**	**443**	**2.56**	**2.45**	**(0.15,0.07)**	**This work**
PPI	3.8	3307	412	0.71	0.40	(0.16,0.06)	66
mTPA-PPI	3.2	4065	404	0.84	0.48	(0.16,0.04)	66
L-BPPI(50nm)	8.5	70	440	0.01	-	(0.16,0.11)	67
L-BPPI(40nm)	6.5	295	440	0.13	-	(0.16,0.11)	67
L-BPPI(30nm)	5.0	420	440	0.40	-	(0.16,0.10)	67
L-BPPI(20nm)	4.5	391	440	0.68	-	(0.16,0.10)	67
Z-BPPI(50nm)	6.5	105	440	0.07	-	(0.17,0.12)	67
Z-BPPI(40nm)	5.0	502	440	0.34	-	(0.16,0.12)	67
Z-BPPI(30nm)	4.5	267	440	0.45	-	(0.16,0.12)	67
Z-BPPI(20nm)	5.0	100	440	0.31	-	(0.16,0.11)	67
TPA-PIM	-	4510	420	1.14	0.79	(0.16,0.04)	68
MADN (BUBD-10%)	7.8	-	440	2.1	-	(0.15,0.10)	69
CPPPI(M1)	-	3322	420	0.65	0.48	(0.16,0.05)	70
PPICPPPI(M2)	-	4329	428	1.53	0.86	(0.16,0.05)	70
PhBPI	2.8	-	450	1.87	1.85	-	71
Trilayer-TPBI	3.0	-	468	2.96	2.00	(0.15,0.19)	72
Bilayer-TPBI	3.2	-	468	2.03	1.00	(0.15,0.15)	72
TPA-BPI	2.8	-	448	2.63	2.53	(0.15,0.09)	73
TPA-BPI	2.8	-	448	1.83	1.58	(0.15,0.09)	73
TPA3TPAN(20nm)	8.9	1867	575	2.45	0.48	-	74
TPA3TPAN(60nm)	8.0	572	579	0.35	0.12	-	74
TPA3TPAN(80nm)	7.4	3101	580	6.16	2.64		74
DTPA4TPAN(20nm)	11.0	387	582	0.35	0.06	-	74
PEDOt-PSS :3(100nm)	4	2800	460	0.61	0.14	(0.15,0.14)	75
PEDOt-PSS :3(50 nm)	3	10600	407	1.68	1.10	(0.16,0.13)	75
PEDOt-PSS :4(40nm)	2.5	21200	392	1.90	1.55	(0.16,0.14)	75
9TPAFSPO	-	-	-	1.50	2.19	(0.16, 0.07)	76
MADN	-	-	-	1.43	0.70	(0.15, 0.10)	76
BDMA	-	-	-	2.20	-	(0.16, 0.12)	77
P2	-	-	-	3.08	1.17	(0.17, 0.19)	78
SPI-TPA	4.5	1050	-	0.43	0.93	(0.18, 0.15)	79

References

[1] J. M. Foster and S. F. Boys, *Rev. Mod. Phys.,* 1960, **32**, 300-302.

[2] S. F. Boys, *Rev. Mod. Phys.,* 1960, **32**, 296-299.

[3] H. Fang, J. Bian, L. Li and W. J. Yang, *Chem. Phys.,* 2004, **120**, 9458-9466.

[4] Q. A. Smith, K. Ruedenberg, M. K. Gordon and L. V. Splipchenko, *J. Chem. Phys.,* 2012, **136**, 244107-12.

[66] Z. Wang, Y. Feng, H. Li, Z. Gao, X. Zhang, P. Lu, P. Chen, Y. Ma and S. Liu, *Phys. Chem. Chem. Phys.,* 2014, **16**, 10837-10843.

[67] W. Li, L. Yao, H. Liu, Z. Wang, S. Zhang, R. Xiao, H. Zhang, P. Lu, B. Yang and Y. Ma, *J. Master. Chem. C,* 2014, **2**, 4733-4736.

[68] M. F. Lin, L. Wang, W. K. Wong, K. W. Cheah, H. L. Tam, M.T. Lee and C. H. Chen, *Appl. Phys. Lett.,* 2006, **89**, 121913-121916.

[69] Z. Gao, Y. Liu, Z. Wang, F. Shen, H. Liu, G. Sun, L. Yao, Y. Lv, P. Lu and Y. Ma, *Chem. Eur. J,* 2013, **19**, 2602-2605.

[70] X. L. Li, X. Ouyang, D. Chen, X. Cai, M. Liu, Z. Ge, Y. Cao and S. J. Su, *Nanotechnology,* 2016, **27**, 124001-11.

[71] Y. Zhang, T. W. Ng, F. Lu, Q. X. Tong, S. L. Lai, M. Y. Chan, H. L. Kwong and C. S. Lee, *Dyes and Pigments,* 2013, **98**, 190-194.

[72] Y. Zhang, S. L. Lai, Q. X. Tong, M. F. Lo, T. W. Ng, M. Y. Chan, Z. C. Wen, J. He, K. S. Jeff, X. L. Tang, W. M. Liu, C. C. Ko, P. F. Wang and C. S. Lee, *Chem. Master.,* 2012, **24**, 61-70.

[73] F. I. Wu, P. I. Shih, M. C. Yuan, A. K. Dixit, C. F. Shu, Z. M. Chung and E. W. G. Diau, *J. Mater. Chem.,* 2005, **15**, 4753-4760.

[74] Y. L. Liao, C. Y. Lin, K. T. Wong, T. H. Hou and W. Y. Hung, *Org. Lett.,* 2007, **9**, 4511-4514.

[75] D. G. Yu, F. C. Zhao, Z. Zhang, C. M. Han, H. Xu, J. Li and et al., *Chem. Commun.,* 2012, **48**, 6157-6159.

[76] M. T. Lee, H. H. Chen, C. H. Liao, C. H. Tsai and C. H. Chen, *Appl. Phys. Lett.,* 2004, **85**, 3301-3303.

[77] J. Y. Park, S. Y. Jung, J. Y. Lee and Y.G. Baek, *Thin Solid Films,* 2008, **516**, 2917-2921.

[78] C. Tang, F. Liu, Y. J. Xia, J. Lin, L. H. Xie, G. Y. Zhong and et al., *Org. Electron.,* 2006, **7**, 155-162.

[79] V. Thanikachalam P. Jeeva and J. Jayabharathi, *J. Phys Org Chem.,* 2017, **3695**, 1-13.
